# Heat Tolerance in Older Adults: A Systematic Review of Thermoregulation, Vulnerability, Environmental Change, and Health Outcomes

**DOI:** 10.3390/healthcare13212785

**Published:** 2025-11-03

**Authors:** Sandra Núñez-Rodríguez, Carla Collazo-Riobó, Javier Sedano, Ana Isabel Sánchez-Iglesias, Josefa González-Santos

**Affiliations:** 1 Faculty of Health Sciences, University Isabel I, 09003 Burgos, Spain; sandra.nunez@ui1.es; 2 Faculty of Health Science, University of Burgos, 09001 Burgos, Spain; ana.sanchez@zamoradipu.es (A.I.S.-I.); mjgonzalez@ubu.es (J.G.-S.); 3 Instituto Tecnológico de Castilla y León, 09001 Burgos, Spain; javier.sedano@itcl.es

**Keywords:** older adults, frailty, thermoregulation, heat stress, systematic review

## Abstract

**Background:** Exposure to heat is a growing health concern in the context of climate change. Older adults (people aged 600 years or older) are particularly vulnerable due to age-related physiological changes that compromise thermoregulation. Objective: To systematically review the evidence on thermoregulatory alterations in older adults exposed to heat and their association with adverse clinical outcomes. **Methods:** Following PRISMA guidelines, a systematic search was conducted in PubMed, Web of Science, ScienceDirect, and Scopus. Twenty-four original studies met the inclusion criteria, including experimental studies in controlled environments and epidemiological studies on heat-related outcomes. Data on study characteristics, thermophysiological responses, clinical outcomes, and methodological quality (assessed with JBI tools) were extracted and synthesized. **Results:** Experimental studies showed that older adults exhibit reduced sweating and cutaneous vasodilation, attenuated cardiovascular and autonomic adjustments, impaired hydration status, and altered thermal perception. These limitations resulted in greater heat storage, faster increases in core temperature, and a higher risk of dehydration and fatigue compared with younger adults. Epidemiological evidence confirmed a significant association between high ambient temperatures and increased hospitalizations and mortality among older populations, particularly at advanced ages, in women, and in those with comorbidities or socioeconomic vulnerability. **Conclusions:** Heat exposure and climatic conditions—particularly high ambient temperatures, humidity, and poor air quality—reduce thermoregulatory efficiency and increase risks of dehydration, cardiovascular strain, and mortality in older adults. Integrated public health actions addressing both environmental and physiological factors are essential for preventing heat-related illness among older adults.

## 1. Introduction

Climate change is driving an increase in the frequency, intensity, and duration of heat waves worldwide [1,2,3]. Prolonged exposure to heat can trigger a series of physiological alterations that, in severe cases, lead to adverse clinical outcomes such as heat stroke, multi-organ failure, and death [4,5]. The World Meteorological Organization has warned that in the coming years, historical temperature thresholds are likely to be repeatedly surpassed, which will further increase the burden of heat-related morbidity and mortality.

Excessive heat represents a significant physiological stressor capable of disrupting multiple body systems [6,7]. When ambient temperature exceeds the body’s capacity for heat dissipation, core temperature rises, initiating a cascade of cardiovascular, sudomotor, and metabolic responses aimed at maintaining thermal homeostasis. If these responses are insufficient or impaired, thermal imbalance ensues, potentially resulting in hyperthermia, multi-organ dysfunction, and, in extreme cases, death [8].

Older adults represent one of the most vulnerable groups to heat exposure, as aging is associated with alterations in thermoregulatory mechanisms, including reduced sudomotor and cutaneous vasodilatory capacity, blunted autonomic and cardiovascular responses, and diminished subjective perception of environmental temperature. These changes limit the ability to dissipate heat and increase the risk of hyperthermia, dehydration, and cardiovascular or respiratory events during intense or prolonged thermal exposures [9,10,11].

This vulnerability is further compounded by the high prevalence of chronic conditions—such as heart failure, coronary artery disease, chronic obstructive pulmonary disease, diabetes mellitus, or dementia—as well as the frequent use of medications that may interfere with sweating, vasodilation, or fluid-electrolyte balance (e.g., diuretics, beta-blockers, anticholinergics) [12,13]. Additional risk factors include reduced thirst sensation, impaired mobility, functional dependency, and limited access to cooler environments [14].

Beyond biological determinants, social and environmental conditions also shape the impact of heat on older populations. Energy poverty, poorly ventilated housing, lack of green spaces, and social isolation may amplify exposure and hinder the adoption of protective measures [15,16]. In urban areas, the “heat island” effect—where cities experience higher temperatures than surrounding rural areas due to structures such as roads, buildings, and pavements that absorb and retain more heat than natural vegetation—can further raise nighttime temperatures, reducing opportunities for physiological recovery [15,16].

Taken together, these biological, clinical, and social determinants of vulnerability converge in the concept of frailty, a multidimensional geriatric syndrome characterized by decreased physiological reserve and impaired homeostatic capacity. Frailty increases susceptibility to stressors such as extreme heat, accelerating functional decline, morbidity, and mortality. Thus, understanding thermoregulatory alterations in the context of frailty is essential to identify at-risk populations and guide preventive and clinical strategies [17,18].

Several reviews have analyzed the effects of heat stress on mortality or morbidity in general populations [19,20,21,22]. However, few have specifically addressed thermoregulatory responses and frailty in older adults. Importantly, previous systematic reviews did not integrate physiological experimental evidence with epidemiological data, leaving a critical gap in understanding the mechanisms that link impaired thermoregulation with adverse clinical outcomes. This systematic review was therefore conducted to synthesize both experimental and population-based studies, focusing on older adults (≥60 years) and their vulnerability to heat exposure.

The aim of this systematic review is to identify and analyze physiological alterations in thermoregulation among older adults (≥60 years) exposed to heat—whether environmental, exercise-induced, or in clinical settings—and their association with adverse clinical outcomes such as hyperthermia, morbidity, hospitalization, or mortality. This review integrates two main perspectives: (a) the physiological dimension, which examines age-related changes in thermoregulation and heat tolerance, and (b) the epidemiological dimension, which explores how climatic factors such as temperature, humidity, and air quality influence morbidity and mortality among older adults.

## 2. Materials and Methods

This systematic review was conducted in accordance with the recommendations of the PRISMA Statement and following the established research protocol. The review protocol was prospectively registered in the International Prospective Register of Systematic Reviews (PROSPERO; ID number: 1162629). The systematic search of scientific literature was conducted between 26 July 2025 and 4 August 2025. To this end, the electronic versions of the PubMed, Scopus, Web of Science, and ScienceDirect databases were consulted.

First, the clinically answerable research question was formulated in PIO format, following the criteria established by Sackett et al. [23]. This can be seen in Table 1.

Next, based on this format and the PIO question, different search strategies were designed and adapted to the different databases consulted. This can be seen in Table 2. Search strategies were tailored to the indexing systems of each database. The WOS interface allowed for a broader and more detailed combination of terms. In contrast, ScienceDirect presented restrictions in the use of Boolean operators and nesting functions, resulting in a necessarily simplified strategy. Scopus permitted a wider combination of terms than ScienceDirect, though still more limited than WOS. These adaptations were necessary to ensure that each database was queried to its maximum potential within its own technical constraints.

Original research studies were selected (a) with an experimental methodological design (controlled trials, laboratory studies) or observational design (cohort, case-control, time series or ecological studies), (b) published in English or Spanish, (c) published since 2015, (d) with at least the abstract available, and (e) that evaluated physiological alterations in thermoregulation (such as body temperature, sweating, skin blood flow, cardiovascular response, autonomic activity, water balance, thermal perception, or cellular markers of heat stress) and/or adverse clinical outcomes (hyperthermia, morbidity, hospitalisation, or mortality) in older adults (≥60 years) exposed to environmental heat, induced by physical exercise or in clinical settings.

Case reports, narrative or systematic reviews, meta-analyses, letters to the editor, or low-quality publications were excluded, as were studies that did not answer the research question, did not include a population over 60 years of age, or exclusively analyzed animals, in vitro models, or pharmacological interventions unrelated to the physiological response to heat.

We limited the search to studies published since 2015 to ensure that the evidence reflects contemporary climate change conditions. In the last decade, the frequency, intensity, and health impacts of heat waves have increased substantially, making earlier data less representative of current risks

In addition, a manual backward search, or snowballing, was conducted to identify potentially relevant studies that had not been captured in the initial search. Sources of gray literature and the reference lists of the selected articles were also reviewed.

Given the substantial methodological heterogeneity among included studies (controlled experiments, ecological studies, case–crossover designs), a meta-analysis was not feasible. Instead, we applied a narrative synthesis approach, grouping studies by design, exposure type, and outcomes.

The selection of articles was performed in two stages using Rayyan 1.4.3 software [24]. In the first stage, titles and abstracts were screened to exclude clearly irrelevant studies. Subsequently, the full texts of potentially eligible articles were assessed. The entire process was carried out independently by two reviewers, and a third evaluator was consulted to resolve any uncertainties or discrepancies.

To ensure consistency in data extraction, a standardized collection sheet was designed, which included the following items: authors, year of publication, country, study design, number and characteristics of participants, variables assessed, measurement instruments used, main findings, and conclusions. Methodological quality and risk of bias of the selected studies were assessed using the Joanna Briggs Institute critical appraisal tools, applying the appropriate version according to study design (controlled trials, cohort, case-control, time series, or ecological studies). A minimum quality threshold of >7 points was established for final inclusion, and a pilot assessment was conducted among reviewers to ensure uniformity in the application of the criteria.

## 3. Results

**Figure 1:**
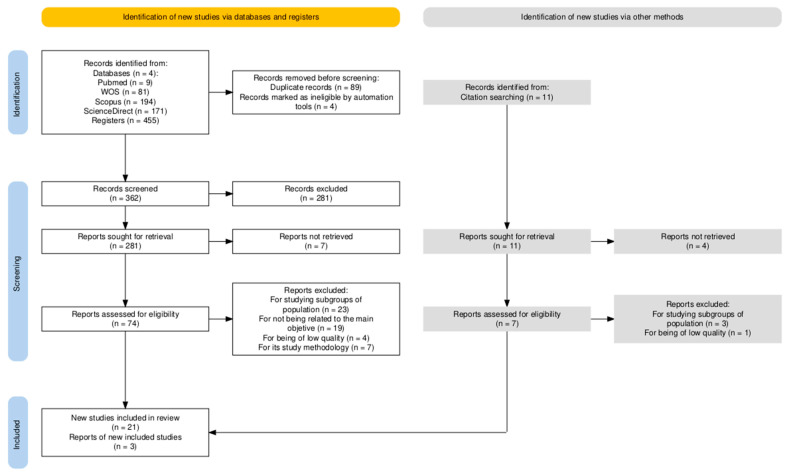
Flow diagram for study selection. Automation tools: automatic filters in databases and reference managers that exclude records with incomplete metadata. Sought for retrieval: label follows PRISMA terminology; 281 records were excluded at title/abstract screening.

A total of 455 results were identified in the initial search. After removal of duplicates (*n* = 89) and automatic exclusion of clearly irrelevant items by database filters and the reference manager (*n* = 4), 362 records were screened by title and abstract. At this stage, 281 records were excluded, leaving 81 reports for retrieval. Seven reports could not be retrieved in full text, so 74 were assessed in detail. Of these, 53 were excluded for predefined reasons, resulting in 21 eligible studies. In addition, a manual backward search (snowballing) was conducted after the database search, reviewing the reference lists of the included studies. This process yielded 11 records, of which 3 were finally included. Figure 1 shows the PRISMA flow diagram outlining the study selection process.

Supplementary Materials include a table summarising the main characteristics and key findings of the included studies, along with their methodological quality scores assessed using the Joanna Briggs Institute (JBI) critical appraisal tool.

### 3.1. Study Characteristics

**Figure 2:**
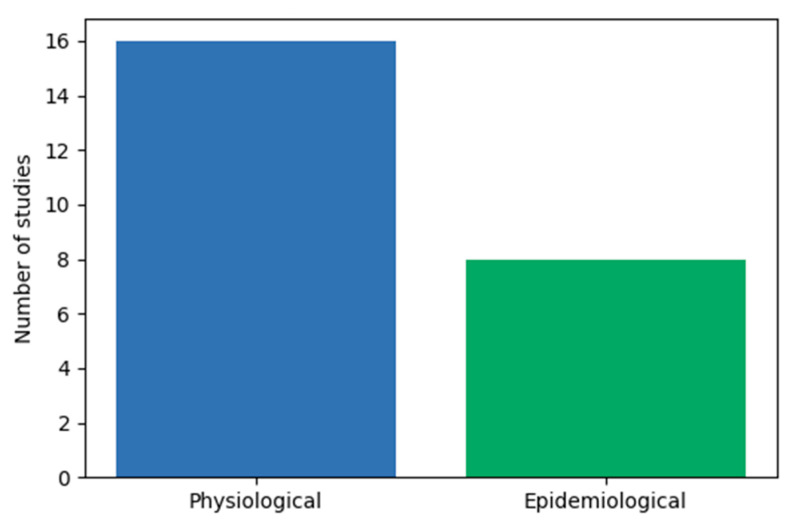
Type of studies involved in this systematic review.

A total of 24 studies were included in the systematic review, of which 16 were non-randomized experimental studies that evaluated thermoregulatory physiology in older adults (≥60 years) under conditions of environmental heat, physical exercise, or controlled thermal exposure [11,25,26,27,28,29,30,31,32,33,34,35,36,37,38,39], and 8 *epidemiological studies* (cross-sectional analytical, time-series, case-crossover, and ecological modeling designs) that examined the relationship between heat exposure and clinical outcomes such as hospitalization, morbidity, and mortality [26,40,41,42,43,44,45,46]. In the epidemiological studies, the association between climatic factors (ambient temperature, humidity, and air quality) and health consequences such as morbidity, hospitalization, and mortality was explicitly evaluated. Figure 2 represents the type of studies involved in a systematic review.

**Figure 3:**
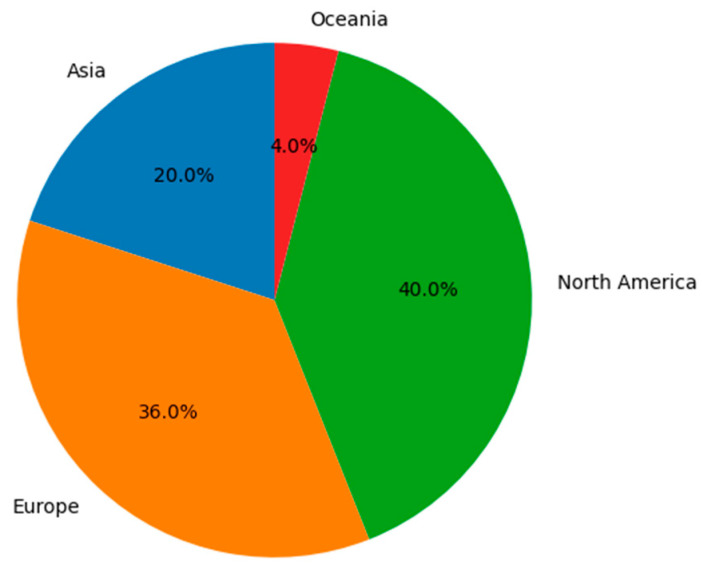
Geographical distribution of the studies.

The geographical distribution of the studies can be seen in Figure 3. The studies were conducted in Asia (*n* = 5, 20.8%) [27,32,45,47,48], Europe (*n* = 9, 37.5%) [7,30,39,41,43,44,45,46,48], North America (*n* = 10, 41.6%) [11,25,27,29,32,33,34,35,37,39], and Oceania (*n* = 1, 4.1%) [31]. Sample sizes in *physiological studies* ranged from 3 to 92 participants, whereas epidemiological studies included from several thousand to more than ten million individuals. The age of older adults ranged from 60 to 83 years in *physiological studies*, while *epidemiological studies* stratified participants into groups of 60–74, ≥75, or ≥85 years. Sex distribution was balanced in most experimental studies, whereas epidemiological research assessed risk differences between men and women.

In *physiological studies*, the most frequently assessed variables included core temperature (measured with rectal thermistors, ingestible capsules, or esophageal probes; *n* = 14) [11,26,27,28,29,30,31,32,33,34,35,36,48,49], mean or local skin temperature (thermocouples, thermistors, or wireless sensors; *n* = 15) [11,26,27,28,29,30,31,32,33,34,35,36,39,48,49], heart rate (ECG, pulse oximeter, or portable monitors; *n* = 15) [11,26,27,28,29,30,31,32,33,36,37,38,39,48,49], blood pressure (automatic sphygmomanometers or photoplethysmography; *n* = 13) [11,27,28,29,30,31,32,35,37,38,45,49,50], sweating and body mass loss (precision scales, ventilated capsules, weight difference calculations; *n* = 10) [11,27,29,30,31,32,34,35,37,38], and cutaneous or muscular blood flow (laser Doppler, Doppler ultrasound; *n* = 7) [11,27,29,31,34,35,37]. Additional parameters such as cardiac output, stroke volume, cutaneous vascular conductance, sympathetic nerve activity (microneurography; *n* = 4) [28,36,37,38], and intramuscular muscle temperature (*n* = 3) [29,37,38] were also measured.

Subjective measures included standardized thermal sensation scales (ASHRAE 7-point scale; *n* = 6) [11,32,33,38,48,49], thermal comfort scales (4–6 point scales; *n* = 5) [11,34,40,49,51], thermal acceptability (*n* = 3) [33,48,49], and environmental symptoms and mood questionnaires (ESQ-IV and POMS-40; *n* = 3). Hydration was evaluated using urinary specific gravity in three studies [11,27,30,38].

*Epidemiological studies* assessed meteorological variables such as mean, minimum, and maximum ambient temperature (*n* = 8) [11,38,39,43,44,45,46,47], relative humidity (*n* = 6) [41,45,46,47,48,50], wind speed (*n* = 3) [47,48,50], or PM_2.5_ pollution (*n* = 2) [47,50]. Outcomes included all-cause mortality (*n* = 6) [41,45,46,47,48,50], cardiovascular mortality (*n* = 4) [45,47,48,50], respiratory mortality (*n* = 3) [45,48,50], and cause-specific hospitalizations or emergency admissions (*n* = 2) [41,46]. Data sources included national or regional mortality and hospitalization registries, with analyses conducted through logistic regression models, generalized additive models, time-series analyses, and distributed lag non-linear models (DLNM).

**Figure 4:**
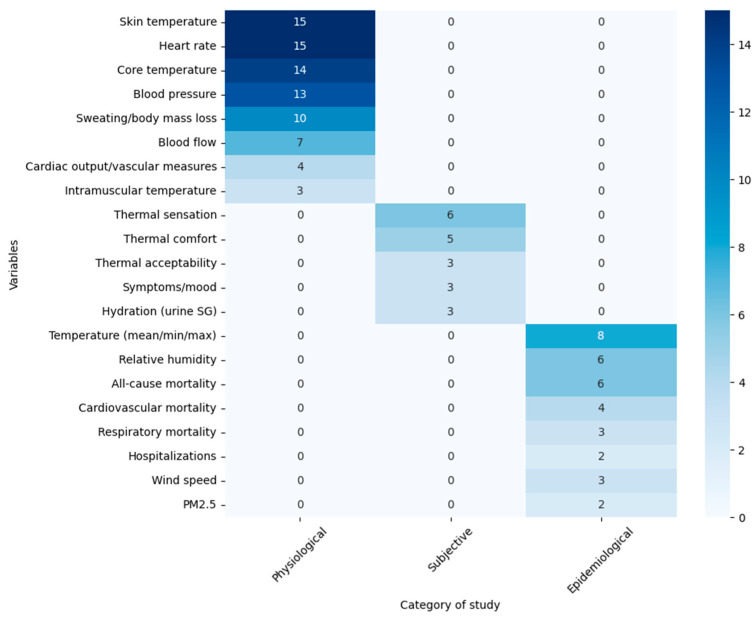
Variables studied during the articles (*n* = 24).

For a clearer understanding of the variables examined in the selected studies, Figure 4 presents each variable together with the article group types and the number of articles in this review that analyze it.

The methodological quality and risk of bias of the included studies were assessed, with all of them obtaining scores above the established cut-off for their design (Table 3, Table 4 and Table 5).

Overall, the methodological quality of the included studies was high. All quasi-experimental and cohort studies achieved scores above 7 on the JBI scale, indicating low risk of bias. The main limitations identified were small sample sizes in some physiological studies and limited control for confounding variables in certain epidemiological analyses. Cross-sectional studies generally demonstrated good internal validity, although external generalizability was occasionally limited due to regional or climatic differences. The consistency across quality indicators supports the reliability of the synthesized findings.

### 3.2. Description of the Characteristics of the Studies

This systematic review encompasses two complementary bodies of evidence. On the one hand, experimental studies in older adults exposed to heat were analyzed, characterizing physiological thermoregulatory responses such as core and skin temperature, sweating, hemodynamics, sympathetic activity, thermal perception and symptoms, hydration/plasma volume, and cellular stress biomarkers. On the other hand, epidemiological studies were included, estimating the risk of mortality and morbidity/hospitalization attributable to these heat-related physiological responses in older populations.

This dual approach makes it possible to link physiological mechanisms observed in laboratory settings with population-level patterns of risk (hyperthermia, hospitalizations, and mortality).

#### 3.2.1. Physiological Alterations in Thermoregulation Among Older Adults Exposed to Heat

Physiological alterations in thermoregulation among older adults exposed to heat were examined in 16 of the 24 articles included in this review. These studies show that older adults accumulate more body heat than younger groups under the same thermal stress, reaching higher final core temperatures and exhibiting more pronounced increases [11,31,48]. This accumulation is accompanied by a reduced ability to dissipate heat, reflected in smaller increases in skin blood flow and sweating [28,29,37,38].

Attenuated cardiovascular and autonomic responses have also been documented, including smaller increases in heart rate, mean arterial pressure, and muscle sympathetic activity during exercise and passive heating compared with young controls [28,30,36].

Regarding thermal perception, older adults display a close relationship between heat sensation and mean skin temperature [32,49], yet some studies report that subjective perception may underestimate actual thermal stress, particularly in individuals with comorbidities such as hypertension or diabetes [27]. This suggests a potential additional risk due to impaired symptom perception.

With respect to hydration, three studies assessed hydration status through urine specific gravity [26,29,36] and one through other indicators such as plasma osmolality and plasma volume changes [11], highlighting that dehydration and fluid losses may contribute to thermoregulatory inefficiency. Body mass loss is also relevant: in simulated heat waves, a ~1.5% reduction in body mass associated with mild dehydration was linked to decreased physical performance and increased perceived fatigue [31].

Finally, some studies have explored cellular markers, showing that prolonged heat exposure in older adults induces increases in proteins related to autophagy (LC3-II and p62 accumulation) and apoptosis, changes attenuated by cooling interventions [34]. These findings highlight that heat responses involve not only thermoregulatory processes but also systemic cellular stress.

#### 3.2.2. Adverse Clinical Outcomes

Population-based studies demonstrate that exposure to high temperatures is significantly associated with increased mortality and hospitalizations in older adults. A U- or J-shaped relationship between temperature and mortality is commonly observed, with risk increasing above thresholds that vary by region and climate [42,43,47]. The risk is greater in advanced age groups (≥75 or ≥85 years) and, in many cases, in women [41,46].

During heat waves, the risk of mortality can rise several-fold compared with neutral temperatures, with the strongest effects on the same day of exposure (lag 0) and, to a lesser extent, in subsequent days [45,47]. Intra-seasonal acclimatization appears to partially mitigate this risk [50] but does not eliminate it during extreme heat events.

Moderate heat, which is more frequent, also contributes substantially to overall mortality in temperate climates [43]. Several studies identify modifying factors such as PM_2.5_ air pollution, which exacerbates cardiovascular mortality risk in older adults during heat waves, and socioeconomic disadvantage [44,45].

Regarding morbidity, elevated temperatures have been linked to an increased risk of hospitalization for specific causes, such as dementia, particularly among individuals aged 75 and older [44]. These effects may occur on the same day of exposure and persist for several days.

Following the synthesis of adverse outcomes, Table 6 summarizes the main physiological and environmental factors influencing heat tolerance in older adults. The table integrates both experimental and epidemiological findings, highlighting mechanisms of thermoregulatory impairment and contextual modifiers such as climate and air quality.

## 4. Discussion

This systematic review was conducted with the primary aim of analyzing physiological alterations in thermoregulation among older adults (≥60 years) exposed to heat—whether environmental, exercise-induced, or in clinical contexts—and their association with adverse clinical outcomes such as hyperthermia, morbidity, and mortality.

Overall, the findings consistently show that older adults exhibit a reduced capacity to maintain thermal homeostasis when exposed to heat, both in experimental settings and during real-world heat waves. Included physiological studies provide evidence of greater core temperature accumulation, diminished sudomotor and vasodilatory responses, and attenuated autonomic profiles—characterized by reductions in sympathetic activity, blood pressure, and heart rate compared to younger adults [11,26,27,28,29,30,31,32,33,35,36,48,49]. These alterations compromise the efficiency of heat dissipation mechanisms, thereby increasing the risk of hyperthermia, dehydration, and cardiovascular collapse.

At the same time, epidemiological evidence complements and reinforces this pattern: multiple studies document significant increases in mortality and hospitalizations among older adults during heat exposure, with greater vulnerability observed at advanced ages (≥75 or ≥85 years) and in women. The U- or J-shaped relationship observed in most contexts reflects that not only extreme temperatures, but also prolonged moderate exposures pose a risk [11,38,39,43,44,45,46,47]. Additionally, comorbidities, socioeconomic disadvantage, and co-exposure to air pollutants such as PM_2.5_ act as modifiers that amplify risk factors that are increasingly prevalent in modern societies [39,44,45,47].

It is also noteworthy that some older adults underestimate their thermal sensation even under high thermal loads, particularly those with hypertension or diabetes [26,31]. This suggests that self-perceived symptoms may not be reliable indicators of risk, complicating the adoption of protective behaviors such as hydration or seeking cooler environments. From a public health perspective, this underscores the need for preventive strategies that do not rely solely on individual self-regulation but instead incorporate early warning systems, safe environments, and community support during heat events.

The interaction between experimental and epidemiological evidence suggests that population-level clinical risk may stem directly from age-related physiological limitations that reduce thermal safety margins. Factors such as body mass and plasma volume loss during heat exposure, along with changes in cellular stress markers, indicate that the impact of heat on older adults may extend beyond acute episodes, potentially producing subclinical or cumulative effects [31,35]. Importantly, the interaction between experimental and epidemiological evidence strengthens the causal inference [6,11,26,27,28,29,30,31,32,33,34,35,36,38,39,43,44,45,48,49]: physiological studies demonstrate specific mechanisms—such as reduced sweat gland output, impaired vasodilation, and diminished cardiovascular responses—that plausibly explain the excess mortality and morbidity observed in population studies. For example, the reduced sudomotor response documented in laboratory settings provides a biological basis for the increased rates of dehydration, syncope, and cardiovascular collapse seen during heat waves. This mechanistic bridge reinforces the interpretation that heat-related deaths among frail older adults are not only statistical associations but have direct physiological underpinnings.

The concept of frailty provides an additional framework for interpreting these findings. Frailty, operationalized through clinical tools such as the Fried phenotype (weakness, slowness, exhaustion, low activity, and weight loss) and the Frailty Index, reflects a reduced physiological reserve and resilience [17,18,51]. Our results suggest that impaired thermoregulatory capacity—especially diminished sudomotor and cardiovascular responses—aligns with these established markers of frailty [25,26,27,28,29,30,31,32,33,34,35,37,38,39,40,41,42,43,44,47,50]. Thus, thermoregulatory failure can be understood as an expression of frailty, amplifying the vulnerability of older adults to heat stress. Linking frailty with thermoregulatory dysfunction not only strengthens the biological plausibility of epidemiological outcomes but also highlights the need to incorporate frailty screening into heat-health prevention strategies.

Furthermore, evidence of plasma volume reduction, impaired cardiovascular control, and altered stress biomarkers indicates that heat exposure may accelerate frailty trajectories over time, moving individuals from a robust state to pre-frailty or frailty [30,32,34,35]. This cumulative perspective bridges acute physiological findings with long-term population health risks.

In terms of implications, the findings support the development of clinical protocols tailored for older adults during heat waves, including objective monitoring (core temperature, hydration status) and preventive strategies such as intermittent cooling, while recognizing that its effects are temporary if exposure persists. Moreover, they reinforce the importance of risk mitigation policies that integrate air pollution control and urban design aimed at reducing heat islands.

A major limitation of this review is the high methodological and population heterogeneity across included studies, which prevented a quantitative synthesis of effect sizes. This variability reflects differences in study designs, environmental exposures, and health outcomes. To address methodological heterogeneity, we synthesized findings narratively, separating experimental and epidemiological evidence. This allowed us to highlight converging results while acknowledging differences in study design and measurement. Another limitation is that most studies originated from high-income countries with temperate climates. This geographical bias limits the extrapolation of the findings to low- and middle-income regions or to regions with more extreme climates. Many experimental studies included small samples with high interindividual variability, limiting generalizability. Epidemiological studies, meanwhile, rely on aggregated data and exposure models that may not accurately reflect individual-level risk. Furthermore, most evidence comes from temperate climates and high-income countries, raising questions about applicability to tropical or low-resource settings.

Future research should prioritize longitudinal studies that integrate physiological measures with clinical outcomes within the same cohorts, as well as evaluations of personalized interventions (e.g., acclimatization programs, optimized cooling strategies) in frail older populations. It will also be essential to investigate how frailty status, polypharmacy, sex differences, and functional capacity shape thermoregulatory responses and health risks, using standardized frailty assessment tools to enable cross-study comparisons.

This review confirms that the vulnerability of older adults to heat results from a complex interaction of physiological changes—some intrinsic to aging—preexisting health conditions, and environmental factors. By explicitly linking frailty constructs with thermoregulatory dysfunction and bridging experimental mechanisms with epidemiological outcomes, our synthesis highlights the need for integrated clinical, public health, and policy approaches to protect older adults during heat stress in a warming climate.

## 5. Conclusions

This systematic review shows that older adults exhibit a reduced physiological capacity to maintain thermal homeostasis during heat exposure, due to impaired sweating, cutaneous vasodilation, and autonomic and cardiovascular responses. These limitations promote greater heat storage and more rapid elevations in core body temperature, increasing the risk of hyperthermia, dehydration, and functional decline. The relationship between impaired thermoregulation and frailty supports established clinical frameworks, reinforcing the importance of identifying frailty as a risk marker for heat-related adverse outcomes. At the population level, consistent increases in mortality and hospitalization among older adults are observed during episodes of high temperatures and heat waves, with heightened vulnerability at advanced ages, in women, and among individuals with comorbidities or social disadvantage.

The findings of this review reinforce the importance of implementing targeted preventive measures for older adults, including clinical surveillance strategies, community support programs, and public policies designed to reduce exposure and enhance resilience to heat. Based on the evidence reviewed, public health policies should prioritize heat-health action plans that integrate environmental monitoring, air-quality improvement, and urban design strategies to reduce heat island effects. Specific measures include community cooling centers, early warning systems for heat waves, and screening of frail older adults to prevent heat-related illness, including systematic screening for early signs of dehydration, heat exhaustion, and heat stroke, alongside initiatives to ensure adequate hydration, access to cooling environments, and strengthened community support for vulnerable older adults during extreme heat events.

## Figures and Tables

**Table 1:** PIO Format.

Population	Older Adults (People Aged 60 Years or Older)
**Intervention**	Exposure to heat (hot environments, ambient heat, physical exercise, heat waves, hospitalization in high temperature conditions)
**Outcomes**	Physiological alterations in thermoregulation (e.g., sweating, vasodilation, body temperature, autonomic dysregulation), and adverse clinical outcomes (such as hyperthermia, morbidity, hospitalization, or mortality)
**Research question**	What physiological alterations in thermoregulation do older adults experience when exposed to heat, and how do these relate to adverse clinical outcomes such as hyperthermia, morbidity, or mortality?

**Table 2:** Search strategy used, adapted to each of the databases.

Base de Datos	Estrategia de Búsqueda
**Pubmed**	(“Aged” [MeSH] OR “Older adults” OR elderly) AND (“Thermoregulation” [MeSH] OR “body temperature regulation”) AND (“Heat stress” [MeSH] OR “hot weather” OR “ambient temperature”) AND (“Sweating” [MeSH] OR “Vasodilation” [MeSH] OR “Blood Flow” [MeSH] OR “core temperature” OR “autonomic function”)
**Scopus**	TITLE-ABS-KEY (elderly OR “older adults” OR aged) AND TITLE-ABS-KEY (thermoregulation OR “body temperature regulation”) AND TITLE-ABS-KEY (heat OR “hot weather” OR “heat stress”) AND TITLE-ABS-KEY (sweating OR “skin blood flow” OR vasodilation OR “core temperature” OR “autonomic function”)
**ScienceDirect**	“older adults” AND thermoregulation AND heat AND (sweating OR vasodilation OR “core temperature”)
**WOS**	TS = (thermoregulation AND (elderly OR “older adults” OR aged) AND (heat OR “heat stress” OR “hot weather”) AND (“sweating” OR “vasodilation” OR “skin blood flow” OR “core temperature” OR “autonomic function”) AND (mortality OR hyperthermia OR “heat-related illness”))

Note: The Web of Science (WOS) database allowed an open-state search strategy, enabling the inclusion of broader combinations of terms.

**Table 3:** Results of the quality assessment of quasi-experimental studies.

	Q1	Q2	Q3	Q4	Q5	Q6	Q7	Q8	Q9
[48]	+	+	+	−	+	+	+	+	+
[26]	+	+	+	−	+	+	+	+	+
[27]	+	+	+	−	+	+	+	+	+
[28]	+	+	+	−	+	+	+	+	+
[29]	+	+	+	−	+	+	+	+	+
[30]	+	+	+	−	+	+	+	+	+
[31]	+	+	+	+	+	+	+	+	+
[32]	+	−	+	+	+	+	+	+	+
[33]	+	+	+	+	+	+	+	+	+
[34]	+	+	+	+	−	+	+	+	+
[35]	+	+	+	+	+	+	+	+	+
[11]	+	+	+	+	+	+	+	+	+
[36]	+	+	+	+	+	+	+	+	+
[37]	+	+	+	−	+	+	+	+	+
[38]	+	+	+	+	+	+	+	+	+

**Table 4:** Results of the quality assessment of cross-sectional studies.

	Q1	Q2	Q3	Q4	Q5	Q6	Q7	Q8
[49]	+	+	+	+	−	−	+	+
[39]	+	+	+	+	+	+	+	+
[46]	+	+	+	+	+	+	+	+

**Table 5:** Results of the quality assessment of cohort studies.

	Q1	Q2	Q3	Q4	Q5	Q6	Q7	Q8	Q9	Q10	Q11
[45]	+	+	+	+	+	+	+	+	+	+	+
[47]	+	+	+	+	+	+	+	+	+	+	+
[44]	+	+	+	+	+	+	+	+	+	+	+
[45]	+	+	+	+	+	+	+	+	+	+	+
[42]	+	+	+	+	+	+	+	+	−	−	+
[41]	+	+	+	+	+	+	+	+	+	−	+

**Table 6:** Factors associated with heat tolerance in older adults.

Category	Factor	Effect on Heat Tolerance	Representative Studies
Physiological	Sweat gland output	Reduces evaporative cooling capacity, leading to greater core temperature accumulation.	[11,26,28,29,30,31,33,36]
	Cutaneous vasodilation	Limits convective heat loss and delays thermoregulatory responses under heat exposure.	[11,27,29,31,34,35,37]
	Plasma volume/dehydration	Increases cardiovascular strain and fatigue, reducing heat dissipation efficiency.	[11,25,28,35]
	Sudomotor and autonomic activity	Reduces sympathetic drive and thermoeffector function during heat exposure.	[27,29,35,36,37]
Epidemiological	Ambient temperature and humidity	Associated with increased hospitalizations and mortality among older adults.	[6,30,37,38,42,43,44,46]
	PM_2.5_ air pollution	Amplifies cardiovascular and respiratory morbidity and mortality during heat waves.	[44,46]
	Socioeconomic disadvantage	Increases exposure, limits access to cooling resources, and reduces adaptive capacity.	[40,43,44]
	Female sex, advanced age (≥85 years)	Associated with higher physiological vulnerability and heat-related deaths.	[40,45,46]
Cross-cutting	Frailty, multimorbidity, polypharmacy	Reduce physiological reserve and thermoregulatory efficiency, heightening susceptibility.	[30,34,47]

Note: Physiological factors were mainly identified in experimental studies, whereas environmental and individual modifiers were derived from epidemiological research.

## Data Availability

No new data were created or analyzed in this study. Data sharing is not applicable to this article.

## Supplementary Materials

The following supporting information can be downloaded at: https://www.mdpi.com/article/10.3390/healthcare13212785/s1. Table S1. Experimental Studies: Characteristics and Main Findings. Table S2. Observational and Epidemiological Studies: Characteristics and Main Findings. Table S3. Observational and Epidemiological Studies: Characteristics and Main Findings.
