# Supplementary material for: Heat Tolerance in Older Adults: A Systematic Review of Thermoregulation, Vulnerability, Environmental Change, and Health Outcomes
Source: Healthcare (Basel). 2025 Nov 3;13(21):2785. doi: 10.3390/healthcare13212785 (PMC12609229; doi:10.3390/healthcare13212785)
Supplement: Supplementary file 1 [file healthcare-13-02785-s001.zip › healthcare-3860067-supplementary.pdf]

SUPPLEMENTARY MATERIALS

Table S1. Experimental Studies: Characteristics and Main Findings.

Study	Typology/Main objetive	Participants	Variables/Instruments	Main findings	International Banking Institute (JBI)
**(50)**	Desing: non-randomised experimental study<br><br>Objetive: Evaluate the thermal perceptions and physiological responses of healthy older adults in conditions of typical thermal changes between neutral and transient/cold/hot environments, to understand their thermal comfort and physiological adaptation to exposure to different temperature intervals (3°C, 5°C, and 6°C).	N= 18<br>Age: 65-83<br>Sex (f/m): 9/9	Ambient temperature: automatic loggers, thermometers.<br>Skin temperature: wireless sensors (7 sites).<br>Tympanic temperature: infrared thermometer.<br>BP & HR: non-invasive physiological monitor.<br>Respiratory rate & Peripheral oxygen saturation (SpO_2): pulse oximeter.<br>Thermal sensation, comfort, acceptability: standardized questionnaires (ASHRAE scales).	Small variations (±3 °C) caused no significant physiological changes and comfort remained high. Larger variations (±5–6 °C) reduced comfort and acceptability; cold raised systolic BP (+10%) and RR (+125%) and decreased HR (−3.3 bpm), while heat reduced systolic (−3.36 mmHg) and diastolic BP (−6.07 mmHg) and RR (−1.84 bpm). Under intense cold, mean skin temperature continued to drop >50 min, tympanic temp fell 0.09–0.24 °C, and BP stayed elevated. Under heat, skin temp stabilized <24 min, BP fell, SpO_2 increased transiently.	8/9
**(27)**	Desing: non-randomised experimental study	N total= 59<br>N young adults: 20	Core temperature: rectal probe / ingestible capsule.<br>Mean skin temperature: 8-site sensors (ISO 9886:2004).	Older adults had +2.4 °C·h higher cumulative core temperature than young ($p<0.001$), with similar skin temperatures (Δ −0.5 °C·h, ns). Hypertensive/diabetic older adults also	8/9

	Objetive: Evaluate thermal and perceptual responses (symptoms and mood) in young adults and older adults during prolonged exposure (9 hours) to heat.	N healthy older adults: 20<br>N older adults with comorbidities: 19<br>Age young adults: 19-31<br>Age older adults: 61-77	Symptoms: Environmental Symptoms Questionnaire (ESQ-IV).<br>Mood: Profile of Mood States (POMS-40).<br>Hydration: urine specific gravity.	accumulated more core heat (+2.3–2.5 °C·h) than young ($p<0.01$). No group differences in total symptoms or heat illness perception; older adults with comorbidities reported 31–36% fewer symptoms and 27–36% lower heat illness perception ($p\leq0.03$). Mood disturbance was lower in older adults (−7%, $p=0.03$), particularly with chronic disease (−10%, $p=0.013$). Women had +3.8 °C·h higher cumulative skin temp than men ($p=0.043$).	
**(28)**	Desing: non-randomised experimental study<br><br>Objective: Establish critical environmental limits for core body temperature (Tc) and heart rate (HR) thresholds in young, middle-aged, and older adults exposed to different heat conditions, to understand how these parameters vary across the adult age range.	Ntotal= 92<br>N young adults: 33<br>N middle adults: 28<br>N older adults: 26<br>Age young adults: 24 ± 3 years<br>Age middle adults: 51 ± 6 years<br>Age older adults: 70 ± 3 años.<br>Sex (f/m): 46/46	Core temp (Tc): telemetry capsules.<br>Skin temp (Tsk): iButton sensors (4 sites).<br>HR: continuous monitoring.<br>Ambient conditions: environmental chamber (Tdb, vapour pressure, RH).<br>Maximal oxygen uptake (VO_2max) & metabolism: open spirometry.<br>Sweat rate & body mass loss: precision scales.	No age differences in metabolic rate or VO_2 in warm-humid ($p\geq0.06$). In hot-dry, sweat rate and mass loss were higher in young ($p=0.03$). Older adults had lower critical Tc limits than middle-aged ($p=0.001$) and young ($p<0.001$) in both environments; age correlated with lower thresholds ($R^2=0.35$–0.43, $p<0.001$). HR increase preceded Tc rise in all groups. Older adults showed HR thresholds at lower temperatures/humidities ($p<0.01$). Tc slope was steeper after inflection ($p<0.0001$).	8/9

**(29)**	Desing: non-randomised experimental study<br><br>Objetive: Investigate whether the response of the cutaneous sympathetic nervous system (SSNA) and cutaneous reflex vasodilation in response to increased core temperature are attenuated in older adults compared to younger adults.	Ntotal= 26<br>N young adults: 13<br>N older adults: 13<br>Age young adults: 23 ± 1 years<br>Age older adults: 67 ± 2 years	Skin temperature (Tsk): Thermocouples (6 sites).<br>Core temp (Tes): Esophageal thermocouple.<br>Cutaneous blood flow: Laser Doppler flowmetry (foot dorsum, 33 °C local heater).<br>BP: Photoplethysmography + automated brachial BP (5 min).<br>HR: Single-lead ECG.<br>Respiration: Strain gauge pneumograph (abdominal).<br>SSNA: Peroneal microneurography (tungsten electrodes).	Baseline oesophageal temperature was similar in young (36.8 ± 0.1 °C) and older adults (36.6 ± 0.1 °C). Passive hyperthermia increased core temperature by ~1.0 °C in both groups. Basal and maximal skin vascular conductance (SVC) did not differ, but the rise in SVC and SSNA during heating was significantly smaller in older adults (%SVCmax $p < 0.001$; SSNA $p = 0.01$). The relationship between ΔSSNA and Cutaneus Vascular Conductance (CVC) remained significant in both groups (young $R^2 = 0.87$; older $R^2 = 0.76$), yet the slope was lower in older adults, indicating reduced vasodilator sensitivity to sympathetic activation. Mental stress elevated SSNA under both thermal and neutral conditions, but changes in BP, HR, and SVC did not differ between age groups. Overall, older adults exhibited preserved baseline vascular conductance but a diminished sympathetic and vasodilator response to heating.	8/9
**(30)**	Desing: non-randomised experimental study<br><br>Objetive: To explore the thermal responses in young and older men to severe passive heating of the lower body that induces total body hyperthermia, and to assess the effect of age	Ntotal= 20<br>N young adults: 11<br>N older adults: 9<br>Age young adults: 21 ± 1 years<br>Age older adults: 69 ± 6<br>Sex (f/m) 0/20	Rectal temp (Tre): Thermocouple (12 cm).<br>Muscle temp (Tmu): Needle microprobe (3.5 cm).<br>Skin temp (Tsk): Thermistors (calf, back, forearm).<br>HR: Polar RCX5 monitor.<br>Gas exchange (VO_2, VCO_2, VE): Mobile spirometry.<br>Physiological Stress Index (PSI): Moran formula (Tre + HR).	Older men had more body fat and lower basal temperatures (rectal, muscle, and skin) than younger men ($P < 0.05$), but did not differ in weight, height, or BMI.<br>The time required to warm up the body was longer in older men (88.2 min vs. 66.3 min; $P < 0.05$), although the total increase in rectal temperature was similar in both groups.<br>Younger men had a higher rectal warming rate and greater increase in heart rate during warming ($P < 0.05$).	8/9

	on the thermoregulatory capacity to transfer heat from the lower body (uncompensable heat condition) to the upper body (compensable condition) and then dissipate heat to the environment.		Sweating & comfort: Scales every 5 min.<br>Hydration/sweat loss: Body mass pre–post.	In older adults, calf muscle and skin temperature increased more, but back and forearm skin temperature increased less ($P < 0.05$).<br>Rectal-to-skin temperature gradients increased more in older adults ($P < 0.05$).<br>Metabolic variables increased in both groups, but were higher in young people ($P < 0.05$).<br>Older people showed a lower physiological stress index, lower sweat loss and lower thermal sensation than young people ($P < 0.05$).<br>The initial increase in rectal temperature was slower in older people (significant after 15 min vs. 5 min in young people).<br>The increase in heart rate was greater in young people (34.2%) than in older people (13.9%) ($P < 0.05$).<br>Only older people showed a post-warm-up increase in rectal temperature (~0.2 °C; $P < 0.05$).	
**(31)**	Desing: non-randomised experimental study<br><br>Objetive: Evaluate whether passive whole-body heat stress reduces arterial stiffness in older adults.	Ntotal= 17<br>N young adults: 8<br>N older adults: 9<br>Age young adults: 26 ± 5 years<br>Age older adults: 70 ± 4<br>Sex (f/m): 7/10	Core temperature: Telemetry pill (HQ Inc., Palmetto, FL, USA)<br>Mean skin temperature: Six thermocouples (Omega Engineering, Stamford, CT, USA)<br>Heart rate: ECG (HP Patient Monitor, Agilent)<br>Body weight: Scale (Health-o-meter Professional Scales, IL, USA)	Core temperature increased with mild/moderate heat stress in both young (36.9→38.2 °C) and older adults (36.9→37.9 °C), with no group difference ($P = 0.37, < 0.01$).<br>Mean skin temperature rose in both ($P < 0.01$) but was higher in older adults during mild stress (+4.4 ± 0.7 °C vs. +3.5 ± 0.7 °C, $P = 0.01$).<br>Total sweat loss and % weight loss were greater in young (0.7 ± 0.2 L; 1.0 ± 0.2%) than older adults (0.4 ± 0.3 L; 0.6 ± 0.4%) ($P = 0.06–0.03$).<br>Heart rate rose in both but less in older adults (mild: +18 ± 4 vs. +29 ± 8 bpm; moderate: +23 ± 5 vs. +38 ± 11 bpm; $P < 0.01$).	8/9

			Blood pressure: Electrosphygmomanometry (Tango+, SunTech)<br>Carotid-femoral pulse wave velocity (cfPWV): Applanation tonometry (SphygmoCor, AtCor Medical)<br>Central aortic pressure waveform: Tonometry + generalized transfer function<br>Aortic pulse/augmentation pressure & index: Derived/calculated from aortic waveform<br>Skin blood flow: Laser Doppler flowmetry (Perimed, Sweden)<br>Cutaneous vascular conductance (CVC): Laser Doppler flux / mean arterial pressure	Brachial BP showed no significant change or group difference (P ≥ 0.22).<br>Brachial pulse pressure increased only in young (+4 ± 6 mmHg, P < 0.01).<br>Older adults had higher resting systolic and aortic pulse pressures (P < 0.01), which decreased with mild/moderate heat (P ≤ 0.04).<br>cfPWV was higher at rest in older adults (P < 0.01) but unchanged with heat (P ≥ 0.49).<br>Baseline cfPWV correlated negatively with its heat-induced change (mild r = −0.56, P = 0.03; moderate r = −0.77, P < 0.01), especially in older adults with higher baseline cfPWV.	
**(32)**	Desing: non-randomised experimental study<br><br>Objetive: Compare physiological responses (heat storage and core temperature) between young adults and older adults during prolonged	Ntotal= 59<br>N young adults: 20<br>N older adults: 39<br>Age young adults: 19-31 years	Total body heat gain, evaporative heat loss, body heat storage: Combined indirect and direct calorimetry<br>Core temperature (rectal): Rectal sensor<br>Mean skin temperature: Skin sensors<br>Cardiac output & stroke volume: Likely Doppler	Older adults had 6 W/m² lower total heat gain and 6 W/m² lower metabolic heat production than young adults (P = 0.001).<br>Evaporative heat loss was initially 18 W/m² lower in older adults (P < 0.001) but equalized after the first hour (P ≥ 0.138).<br>They stored 11 W/m² more heat (+88 kJ in 3 h; P = 0.002–0.006).<br>Core temperature rose by 0.2 °C between hours 3–6 in older adults (P < 0.001) vs. no change in young (P	9/9

	exposure (9 hours) to extreme dry heat, simulating heatwave conditions.	Age older adults: 61-78 years<br>Sex (f/m): 22/37	echocardiography, thermodilution, or bioimpedance<br>Forearm blood flow: Likely Doppler ultrasound or plethysmography<br>Blood pressure (SBP/DBP): Standard sphygmomanometer<br>Heart rate: Heart rate monitor<br>Rate pressure product: Calculated from HR and BP<br>Plasma volume change: Blood analysis (hematocrit/protein)<br>Body weight: Precision scale	≥ 0.216), reaching 0.3 °C higher at 6 h ($P < 0.001$).<br>Mean skin temperature showed no group difference ($P = 0.176$).<br>Cardiac output was 1.0 L/min lower and forearm blood flow 1.3 mL/100 mL/min lower in older adults ($P \leq 0.004$).<br>They had 4% greater plasma volume reduction ($P = 0.002$) with no difference in body weight loss ($P = 0.971$).<br>Women showed lower heat gain (−5 W/m^2) and loss (−10 W/m^2) but stored 5 W/m^2 more heat and had +0.4 °C higher skin temperature ($P \leq 0.005$).<br>Each +10 years of age increased core temperature by +0.1 °C ($P < 0.002$), rising to +0.7 °C/10 years in those with type 2 diabetes and/or hypertension ($P < 0.001$), with no change in those without comorbidities.	
**(33)**	Desing: non-randomised experimental study<br>Objetive: Analyse thermal sensations and physiological responses in participants of different ages exposed to a non-uniform thermal environment while bathing, to understand how age affects thermal perception and bodily	Ntotal:3<br>N young adult: 1<br>N middle adult: 1<br>N older adult: 1<br>Age young adult: 25 years<br>Age middle adult: 52 years<br>Age older adult: 71 years<br>Sex (f/m): 0/3	Air temperature: Onset HOBO temp/RH logger UX100-011A<br>Relative humidity: RH sensor (1–95%)<br>Skin temperature: Graphtec Midi logger GL840 (T-type thermocouple)<br>Core temperature: N543 8CH data logger (thermistor)<br>Blood pressure (BP): OMRON Automatic Sphygmomanometer HEM-7200<br>Heart rate (HR): Heart rate monitor (40–180 bpm)	During bathing, wind chill (WC) rose rapidly to +3 in the 25- and 52-year-olds but only to +2 and more slowly in the 71-year-old.<br>The mean skin temperature of the older participant was 1.16 °C lower, especially in air-exposed areas.<br>Core (tympanic) temperature in younger adults fell initially then rose by ~1 °C within 35 min, while in the older participant the fall was smaller and the rise delayed.<br>Diastolic BP decreased by 14 mmHg (52 y) and 17 mmHg (71 y).<br>HR increased in all (×1.39, ×1.21, and ×1.32 for ages 25, 52, and 71 y, respectively).<br>Stroke volume and cardiac output rose more in the	8/9

	responses under these conditions.		Blood flow (BF): Ultrasound imaging (Viamo™ c100, Canon)<br>Oxygen saturation (SpO_2): OMRON P300 Intelli IT pulse oximeter<br>Body weight: Mettler Toledo scale 1d1<br>Thermal sensation (TS): ASHRAE 7-point scale (−3 cold to +3 hot)	young, with CO increasing 2.3×, 1.6×, and 1.4× in the 25-, 52-, and 71-year-olds.<br>Lower-limb blood flow increased markedly (+892 mL/min in the 25-year-old vs. ~+345 mL/min in the others).<br>SpO_2 remained stable (95–98%) in all.<br>All participants lost body weight after bathing, greatest in the 25-year-old (−0.246 kg), indicating water loss.	
**(34)**	Desing: non-randomised experimental study<br><br>Objetive: Evaluate the perceptual responses (environmental symptoms and mood) of older adults subjected to prolonged exposure to simulated environmental heat with or without cooling intervention.	Ntotal: 40<br>Age: 64-79 years<br>Sex (f/m): 16/24	Core temperature: Rectal probe or ingestible VitalSense® capsule<br>Skin temperature: Wireless sensors at 8 body sites (mean calculated per ISO 9886:2004)<br>Subjective environmental symptoms: Environmental Symptoms Questionnaire (ESQ-IV)<br>Mood state: Profile of Mood States (POMS-40)<br>Heart rate (HR): Holter monitor<br>Blood pressure (BP): Manual brachial measurement	At 3 h, no group differences in skin or core temperature ($p > 0.999$).<br>At 6 h, the cooling group showed significantly lower skin and core temperatures than controls ($p < 0.001$), with differences disappearing by hour 9 ($p > 0.999$).<br>AUC for skin and core temperature was 4.0°C·h and 1.6°C·h lower in the cooling group ($p = 0.047$; $p = 0.010$).<br>HR rose in both groups ($p < 0.001$) but was lower at hour 6 in the cooling group ($p < 0.001$), returning to baseline by hour 9.<br>Mean BP showed no between-group differences ($p > 0.636$).<br>At the end of warming, total symptom score (fatigue, headache, dizziness, nausea, sweating, weakness) was 42% lower in the cooling group (0.58×; $p < 0.001$).<br>Symptoms correlated positively with skin temperature in controls but not in the cooling	9/9

				group (interaction $p < 0.001$).<br>Perceived heat illness was 44% lower (0.56×; $p < 0.001$).<br>Cooling participants reported fewer negative and more positive sensations (freshness, comfort, well-being).<br>Total mood disturbance was 9% lower (0.91×; $p = 0.036$), remaining significant after adjusting for core temperature ($p = 0.022$) but not for skin temperature ($p = 0.074$).<br>Energy index: no significant differences ($p > 0.14$).	
**(35)**	Desing: non-randomised experimental study<br><br>Objetive: To evaluate the effect of cooling intervention during a prolonged heatwave on intracellular markers of autophagy, apoptosis, heat shock response, and acute inflammation in older adults, adjusting for the individual degree of body temperature increase during exposure.	N: 38<br>Age: 64-79 years<br>Sex (f/m): 16/20	Core temperature (rectal): Mon-a-therm thermocouple (Mallinckrodt Medical Inc.)<br>Core temperature (ingestible): VitalSense capsule (Mini Mitter Co.)<br>Whole-body heat storage: Direct + evaporative calorimetry (Snellen air calorimeter)<br>Whole-body fluid loss: Change in body mass (CBU150X scale, Mettler Toledo)<br>Plasma volume change: Hemoglobin and hematocrit (Ac·T diff analyzer, Beckman Coulter)<br>Autophagy proteins (LC3-II, p62, LC3-II/I, ULK1, beclin-2): Western blot in PBMCs	Before cooling, core temperature rose similarly in both groups (+0.8 °C vs. +0.9 °C) and HR increased by ~+16–17 bpm.<br>A 2-h cooling phase reduced core temperature by −0.8 °C (95% CI −0.9, −0.6; $P < 0.001$) and HR by −10 bpm (95% CI −15, −6; $P < 0.001$) vs. control; differences disappeared after re-heating.<br>In controls, LC3-II and p62 increased 1.4× and 1.6× from baseline ($P < 0.001$), whereas cooling prevented these increases (p62 slightly ↓; $P = 0.048$).<br>Between-group comparisons showed a 1.9× higher LC3-II (95% CI 1.2–2.8; $P = 0.005$) and 2.9× higher p62 (95% CI 1.4–3.8; $P = 0.002$) in controls vs. cooling.<br>Cleaved caspase-3 rose 1.8× above baseline in controls ($P < 0.001$), 2.8× greater than in the cooling group (95% CI 1.6–4.7; $P < 0.001$), indicating higher apoptosis activation.<br>No group differences in pro-caspase-3, TNF-α, IL-6, HSP70, or HSP90 ($P \geq 0.20$).	8/9

			(Histopaque-1077, Sigma-Aldrich)<br>Apoptosis proteins (cleaved/pro-caspase-3): Western blot in PBMCs<br>Heat shock proteins (HSP70, HSP90): Western blot in PBMCs<br>Cytokines (TNF-α, IL-6): Western blot in PBMCs	Body mass and plasma volume losses were similar and not associated with molecular responses.	
**(36)**	Desing: non-randomised experimental study<br><br>Objetive: To evaluate the effect of cooling intervention during a prolonged heatwave on intracellular markers of autophagy, apoptosis, heat shock response, and acute inflammation in older adults, adjusting for the individual degree of body temperature increase during exposure.	Ntotal= 24<br>N young adults: 12<br>N older adults: 12<br>Age young adults: 22 ± 3 years<br>Age older adults: 59 ± 4 years<br>Sex (f/m): 0/24	Core temperature: Rectal probe (Mallinckrodt Medical, MO, USA), continuous monitoring<br>Serum irisin: ELISA (DY9420-05, DY008; Bio-Techne, ON, Canada), plate reader (Synergy, Biotek, VA, USA)<br>VO_2max: Incremental cycling test (CSEP, 1986) using Medgraphics Ultima Series (MGC Diagnostics, MN, USA)<br>Environmental conditions: Controlled chamber; WBGT 16 °C (temperate) and 32 °C (hot)<br>Exercise duration: Recorded until voluntary stop or safety temperature limit reached<br>.	All participants completed 180 min of exercise in temperate conditions; in heat, 7 young and 5 older adults stopped early. Exercise duration did not differ between groups (young: 180 [96–180] min; older: 170 [78–180] min; $p > 0.05$).<br>Core temperature rose above baseline after exercise in both conditions ($p < 0.001$) but remained elevated only in older adults during recovery in heat ($p = 0.006$).<br>Post-exercise and recovery core temperature were higher in hot vs. temperate conditions for both groups ($p < 0.001$), with no age-group difference.<br>Serum irisin levels were consistently higher in young adults at all time points ($p < 0.01$).<br>In temperate conditions, irisin did not change significantly; in heat, it increased during exercise and recovery in both groups ($p \leq 0.043$).<br>While post-exercise irisin variation was similar across conditions, its recovery elevation was greater in heat than in temperate conditions for both groups.	9/9

**(37)**	Desing: non-randomised experimental study<br><br>Objetive: Compare physiological responses to extreme heat between young adults (18–39 years) and older adults (≥65 years) including light physical activities to simulate activities of daily living, with an emphasis on thermoregulation and risk of hyperthermia.	N total: 40<br>N young adults: 20<br>N older adults: 20<br>Age young adults: 18-39 years<br>Age older adults: ≥65 years<br>Sex (f/m): 20/20	Core temperature: Rectal thermocouple (Mon-a-therm) or telemetric pill (e-Celsius, BodyCap)<br>Skin temperature (Tskin): Local thermocouples on chest, back, abdomen, thigh, calf<br>Heart rate: Six-lead ECG (GE Medical Systems)<br>Blood pressure: Automated sphygmomanometer (Tango+, SunTech)<br>Forearm blood flow: Duplex ultrasound (iE33/EPIQ 7, Philips)<br>Local sweat rate: Ventilated capsule with N_2 + capacitance hygrometry (Vaisala)<br>Total sweat / body mass loss: Precision scale (Mettler Toledo)<br>Thermal perception: 0–8 scale<br>Environmental stress symptoms: Modified Environmental Symptom Questionnaire<br>Plasma / blood volume: Hematocrit (microcapillary), hemoglobin (ABL90 Flex), plasma osmolality (Abbott Alinity)	Core temperature: ΔT core was greater in older adults (1.37 ± 0.42°C) than younger adults (0.68 ± 0.27°C; P < 0.001, d = 1.94). Final core temperature higher in older adults (38.15 ± 0.43°C vs. 37.81 ± 0.26°C; P = 0.005, d = 0.95).<br>Sweat and blood flow: Absolute sweating rates and forearm blood flow were similar, but indexed by ΔT, older adults showed lower sweating (P = 0.003, d = 0.96) and lower forearm blood flow (P < 0.001, d = 1.45).<br>Heart rate & BP: Older adults had a greater increase in heart rate (interaction P = 0.039); mean arterial pressure decreased similarly in both groups.<br>Plasma osmolality: Baseline osmolality higher in older adults (P = 0.014), final osmolality, body mass loss, and plasma volume changes did not differ.<br>Secondary findings: Another dataset confirmed higher ΔT core in older adults (1.02 ± 0.32°C vs. 0.58 ± 0.25°C; P < 0.001, d = 1.52) without significant difference in final core temperature (37.83 ± 0.35°C vs. 37.67 ± 0.34°C; P = 0.151). Indexed sweat and blood flow responses were still lower in older adults (sweat P = 0.002, d = 1.01; blood flow P < 0.001, d = 1.29). Basal and final plasma osmolality remained higher in older adults (P ≤ 0.041), with no differences in osmolality changes, body mass loss, or plasma volume.	9/9

			Metabolic heat production: Indirect calorimetry (PARVO Medics True-One)		
**(38)**	Desing: non-randomised experimental study<br><br>Objetive: Evaluate how age affects cardiovascular and neurovegetative response (Muscle Sympathetic NErve Activity -MSNA-, blood pressure, heart rate) during controlled exposures to passive heat, cold water immersion test (CPT) and lower body negative pressure (LBNP).	N total= 21<br>N young adults: 11<br>N older adults: 10<br>Age young adults: 28 ± 4 years<br>Age older adults: 70 ± 5 years<br>Sex (f/m): 14/7	Core temperature: Ingestible telemetric pill<br>Mean skin temperature: Thermocouples on abdomen, calf, chest, lower back, thigh, upper back; weighted average<br>Heart rate (HR): ECG + cardiotachometer<br>Blood pressure (SBP, DBP, Mean Arterial Pressure -MAP-): Brachial auscultation (Tango+)<br>Muscle sympathetic nerve activity (MSNA): Tungsten microelectrode in peroneal nerve<br>Plasma catecholamines (noradrenaline, adrenaline): Venous blood, HPLC<br>Urine specific gravity (hydration): Handheld refractometer	Baseline comparisons: Core temperature (Y: 37.1 ± 0.2°C vs. O: 36.9 ± 0.2°C, P = 0.45) and mean skin temperature (Y: 34.8 ± 0.3°C vs. O: 34.6 ± 0.7°C, P = 0.33) were similar; mean blood pressure higher in older adults (Y: 86 ± 6 vs. O: 96 ± 5 mmHg, P ≤ 0.02).<br>Sympathetic activity: MSNA higher in older adults—burst frequency (Y: 15 ± 4 vs. O: 31 ± 3 bursts/min, P ≤ 0.01) and burst incidence (Y: 26 ± 8 vs. O: 50 ± 7 bursts/100 CC, P ≤ 0.01); plasma noradrenaline higher in older adults (Y: 145 ± 22 vs. O: 288 ± 51 pg/ml, P ≤ 0.01), plasma adrenaline unchanged (P = 0.62).<br>Passive heat exposure: Core and skin temperature increased similarly in both groups (core +0.6°C, +1.2°C, P ≥ 0.35); heart rate increased less in older adults (e.g., +41 ± 8 vs. +27 ± 6 bpm at +1.2°C, P ≤ 0.01); mean arterial pressure decreased in both groups (P ≤ 0.01), no age differences (P = 0.55). MSNA and noradrenaline increased with heat (P ≤ 0.01), adrenaline unchanged.<br>Additional stress tests: Cold pressor test (CPT) over heat increased MAP (less in older adults, P ≤ 0.02), MSNA and catecholamines (P ≤ 0.01), no age differences in MSNA/catecholamines. Lower Body Negative Pressure -LBNP- 15 mmHg over heat increased heart rate (P ≤ 0.01), no changes in blood pressure (P = 0.67), no age differences.	9/9

**(39)**	Desing: non-randomised experimental study<br><br>Objetive: Evaluate how age affects cardiovascular and neurovegetative response (MSNA, blood pressure, heart rate) during controlled exposures to passive heat, cold water immersion test (CPT) and lower body negative pressure (LBNP).	N= 20<br>N young adults: 11<br>N older adults: 9<br>Age young adults: 19-21 years<br>Age older adults: 64-80 years<br>Sex (f/m): 0/20	Anthropometrics & Body composition: Tanita TBF-300, skinfold calipers (SH5020)<br>Rectal temperature: Thermistor thermocouple (Ellab, ±0.01 °C)<br>Muscle temperature: Needle microprobe (Ellab)<br>Skin temperature: Thermistors (Ellab, ±0.01 °C)<br>Torque & muscle function: Isokinetic dynamometer (Biodex System 4), electrical stimulator, surface electrodes<br>Muscle activity & reflexes: Bipolar Ag–AgCl electrodes, EMG software (DataLog), tibial nerve stimulation	Body composition: Older adults had higher body fat and subcutaneous fat ($p < 0.05$); no differences in body mass, height, BMI, or surface area.<br>Temperature responses: Lower body warming took longer in older adults (88.2 ± 5.4 min vs. 66.3 ± 6.4 min; $p < 0.05$), though rectal temperature increase was similar (~2.4 °C). Calf muscle temperature increased more in older adults (+5.10 ± 0.18 °C vs. +3.99 ± 0.14 °C, $p < 0.05$), as did skin temperature (+9.92 ± 0.62 °C vs. +7.65 ± 0.33 °C, $p < 0.05$).<br>Reflex & EMG: At rest, older adults had lower Hmax, Vsup, Mmax amplitudes and Hmax/Mmax ratio ($p < 0.05$). Warming reduced Hmax and Vsup in both groups; latencies were longer in older adults and decreased more after warming. Older adults had lower RMS and higher mean frequency (MnF) at baseline; warm-up reduced RMS and increased MnF similarly in both groups.<br>Muscle torque: Before warming, older adults had lower voluntary and electrically induced torque, slower contractions (PTT100), and longer relaxations. After warming, older adults showed greater improvements in induced torque (1 Hz P1, TT-100) and contraction speed (–13.86 ± 1.57 % vs. –8.38 ± 1.81 %, $p < 0.05$). Maximum voluntary activation (CAR) was unchanged by warming.	8/9
**(40)**	Desing: non-randomised experimental study	N= 14<br>Age: 62 ± 7 years<br>Sex (f/m): 5/9	Core and skin temperatures: Ingestible telemetric pill (core), six skin thermocouples (Tsk),	Mild whole-body heating (WBH, ~21 min, Tsk > 35.5 °C, ΔTcore < 0.3 °C):<br>• SBP, DBP, MAP, MSNA lower than baseline; Heart Rate (HR) higher ($p < 0.05$)	9/9

Objetive: To evaluate whether moderate whole-body warming or local forearm warming attenuates muscle sympathetic nerve activity (MSNA) and cardiovascular responses (mean arterial pressure, heart rate) during isometric exercise and Post-Exercise circulatory Oclussion (PECO) in healthy older adults.	mean body temperature calculated as 0.9 Tcore + 0.1 Tsk<br>Forearm measures: Muscle temperature (needle microprobe), skin temperature (adjacent thermocouple), skin blood flow (Laser-Doppler), cutaneous vascular conductance, sweat rate (ventilated capsule)<br>Cardiovascular: Systolic, diastolic, mean arterial pressure (automated sphygmomanometer), beat-by-beat blood pressure (BP) (Finometer), heart rate (ECG), cardiac output and stroke volume (transthoracic echocardiography + tissue Doppler)<br>Autonomic: Muscle sympathetic nerve activity (MSNA, peroneal nerve microelectrode)<br>Respiratory: Piezoelectric pneumography	• MSNA burst rate decreased in older adults (Δ −7.9 ± 6.0 bursts/min) but increased slightly in young adults (Δ 2.7 ± 6.6, $p = 0.01$)<br>Moderate WBH prior to exercise (ΔTcore +0.39 ± 0.13 °C):<br>• DBP and MAP lower, HR, skin blood flow (SkBF), and cutaneous vascular conductance (CVC) higher than normothermic baseline ($p < 0.05$)<br>• MSNA and SBP unchanged<br>• Cardiac output increased; stroke volume unchanged<br>Exercise, PECO, and passive stretching:<br>• Increased MSNA, HR, and MAP in both WBH and normothermic conditions ($p < 0.05$)<br>• In WBH, absolute HR higher and MAP lower than normothermic control ($p < 0.05$)<br>• Last minute of exercise: Older adults had smaller increases in MAP (Δ 16.3 ± 7.5 vs. 23.4 ± 5.6 mmHg, $p = 0.007$), MSNA (Δ 8.4 ± 6.5 vs. 17.9 ± 11.4 bursts/min, $p = 0.03$), and HR (Δ 8.7 ± 5.7 vs. 22.7 ± 9.8 bpm, $p < 0.001$) compared with young adults under WBH<br>• Metaboreceptor stimulation (PECO) + passive stretching: MAP lower, HR higher than normothermic; MAP and MSNA responses greater during PECO + stretching than PECO alone ($p < 0.05$)

				• Older adults had smaller MSNA (Δ 8.7 ± 6.7 vs. 17.3 ± 13.0 bursts/min, p = 0.046) and HR (Δ 1.6 ± 2.7 vs. 10.0 ± 7.8 bpm, p < 0.001) responses than young adults during PECO	
**(43)**	Desing: observational epidemiological<br><br>Objetive: Estimate the sex- and age-specific mortality associated with the record temperatures recorded between 30 May and 4 September 2022, and compare it with the summer of 2003 and the warming trend observed in Europe during 2013–2022.	N= 38.881<br>Age: 0-64 / 65-79 / > 80 years	Global temperature trends: historical records, climate monitoring datasets<br>Heat waves: identified via daily/weekly temperature anomalies<br>Heat-related mortality: national/regional mortality records, Eurostat, epidemiological models estimating relative risk (RR) by age, gender, and location	The optimum temperature for minimum mortality was approximately 17–19 °C, higher in older adults and slightly higher in men than in women.<br>Heat-related mortality increased with age, being most pronounced in women aged 80+ years and in younger men (0–64 years) during heat waves.<br>Total mortality during the summer of 2022 reached 61,672 deaths (95% CI: 37,643–86,807), with a higher number of deaths in women (35,406; 95% CI: 21,576–46,634) compared to men (21,667; 95% CI: 14,684–27,998).<br>Mortality rose with age: 0–64 years: 4,822 deaths; 65–79 years: 9,226 deaths; 80+ years: 36,848 deaths.<br>The countries with the highest number of heat-related deaths were Italy (18,010), Spain (11,324), Germany (8,173), France (4,807), the United Kingdom (3,469), and Greece (3,092).<br>Mortality rates per million population also showed marked increases: 0–64 years: 16; 65–79 years: 160; 80+ years: 1,684; total Europe: 114 (95% CI: 69–160) per million.<br>The highest heat-related mortality rates were observed in Mediterranean countries: Italy 295/million, Greece 280/million, Spain 237/million, and Portugal 211/million.	10/11

Table S2. Observational and Epidemiological Studies: Characteristics and Main Findings

Study	Typology/Main objetive	Participants	Variables/Instruments	Main findings	International Banking Institute (JBI)
(22)	Desing: Analytical cross-sectional study<br><br>Objetive: To evaluate the impact of transient temperature changes during the process of getting up in winter on the thermal physiology and thermal perception of older adults, compared to young adults.	N= 35<br>N young adults: 5<br>N older adults: 30<br>Age young adults: 20-30 years<br>Age older adults: ≥60 years<br>Sex (f/m): 18/17	Outdoor & indoor air temperature (Ta) and relative humidity (RH): Thermometers & hygrometers<br>Cover temperature (Tcover): Thermometer under quilt<br>Black globe temperature (Tg): Black globe thermometer<br>Heart rate (HR) & blood pressure (BP): Physiological monitoring wristband<br>Skin temperature (Tsk): Button thermometers on forehead, chest, arm, hand, thigh, foot<br>Core temperature (Tcore): In-ear thermometer<br>Thermal sensation & comfort: Questionnaires (-3 to +3 scale)<br>Thermal preference: Questionnaire (-1 to +1 scale)	Older adults showed a sharp increase in heart rate upon waking (from 68 to 88 bpm), followed by a drop to 76 bpm, with greater variability and amplitude than young adults, despite lower resting HR. 25% were at risk of tachycardia associated with temperature differences >15 °C, and HR variability increased with BMI, being higher in obese individuals.<br>Blood pressure showed small fluctuations, with 5% at risk of borderline hypertension, without significant influence from temperature differences. Core body temperature remained stable during getting up, although clothing thermal resistance was higher in older adults. Skin temperature in exposed extremities decreased more in older adults, particularly in hands and feet, indicating reduced thermal adaptation.<br>Overall thermal perception was warm, with lower skin temperature sensitivity than young adults, who were more sensitive in chest and legs. Gender differences in local thermal perception were observed in young adults: men more sensitive in legs, women in chest.<br>Thermal perception in older adults was weakly influenced by mean skin temperature, unlike the stronger relationship seen in young people.	6/8

(31)	Desing: Analytical cross-sectional study<br><br>Objetive: Investigate skin temperature (Tsk) at rest and after acute exercise in older adults, and evaluate the influence of gender and cardiorespiratory fitness (CRF) on Tsk.	N: 92<br>Age: 65-75<br>Sex (f/m): 41/51	Skin Temperature (Tsk): Infrared thermography (FLIR E60), 25 ROIs.<br>Cardiorespiratory Fitness (CRF): Graded exercise test (modified Bruce protocol, Lode Valiant treadmill); VO2peak (ml/min, ml/kg/min).<br>Body Composition: Multi-frequency bioelectrical impedance (Tanita MC-780MA); body mass, BMI, fat mass, fat-free mass.<br>Anthropometry: Height (SECA 225).	Women had higher absolute/relative fat and lower CRF; BMI similar between sexes.<br>Men had higher resting Tsk in 19/25 ROIs; largest differences in posterior thigh (+0.91 °C), posterior calf (+0.90 °C), posterior arm (+0.80 °C), anterior thigh (+0.60 °C). Most differences remained after adjusting for fat mass and VO2peak, except chin.<br>After exercise: distal upper extremities (hands/fingers) increased in temperature; proximal upper extremities (arms/torso) decreased; lower extremities increased; core stable. Men had larger forearm temperature decreases than women; differences in posterior arm, posterior leg, anterior thigh remained significant after adjustment.<br>Higher VO2peak associated with greater lower extremity temperature increases in both sexes; men had lower proximal upper extremity temperature, women had lower core and higher distal upper extremity temperatures. Similar patterns observed using time to exhaustion.	8/8
(32)	Desing: case-crossover<br><br>Objetive: Evaluate the intra-seasonal variation (beginning vs. end of the warm season) in the association between exposure to high ambient temperatures and the risk of mortality, and identify population subgroups	N: 1.132.980<br>Age: ≤64 / 65–74 / ≥75 years<br>Sex (f/m): 479.121/ 653.859	Daily Temperature, Relative Humidity, Wind Speed: China Meteorological Data Network (0.01° × 0.01° resolution).<br>Daily $PM_{2.5}$ Concentration: Validated predictive model (0.01° × 0.01°).<br>Mortality Data (all-cause and cause-specific, ICD-10): Official Shandong CDC registry,	Warm season (June–September) daily temperature averaged 24.1 ± 3.3 °C; extreme heat defined as >36.6 °C.<br>1,132,980 deaths recorded in 1,822 subdistricts (2013–2018).<br>J-shaped relationship between temperature and mortality; MMT = 22.5 °C.<br>Extreme heat increased mortality risk: cumulative OR 3.41 (95% CI: 3.11–3.74) over 0–10 days; effect strongest on same day (lag 0), decreasing to day 6, with mortality deficit until day 9.	11/11

	that are most vulnerable to extreme heat.		including date of death, age, sex, education, residential address.<br>Mortality Risk Estimation: Conditional logistic regression with distributed lag non-linear models (DLNM), OR at 97.5th percentile vs. minimum mortality temperature (MMT).	Risk decreased across the season: early warm season OR 3.52 → late warm season OR 2.85 (p = 0.028).<br>Vulnerable groups: women (OR 4.03 vs. 3.00 for men, p = 0.002), adults >75 years (OR 5.06 vs. 2.14 for <65, p < 0.001), lower education (OR 3.43 vs. 1.79 for higher education, p = 0.003).<br>Major causes of heat-related mortality: cardiovascular (OR 5.35) and respiratory diseases (OR 4.73).<br>No significant urban–rural differences (OR 3.23 vs. 3.47, p = 0.450).	
(33)	Desing: ecological observational time series<br><br>Objetive: Estimate heat-attributable mortality in Cyprus for future decades of the 21st century under moderate (SSP2-4.5) and extreme (SSP5-8.5) climate scenarios, based on the observed relationship between ambient temperature and mortality in the period 2004–2019.	N= 53.964<br>Age: < 65 years / > 65 years<br>Sex (f/m): 26.331/27.633	Daily Cause-Specific Mortality: Daily counts by cause from Cyprus Ministry of Health (Health Monitoring Unit), including date, sex, age, cause of death (ICD-10).<br>Daily Ambient Temperature: Mean of daily minimum and maximum from 7 Cyprus Meteorology Department stations.<br>Daily Relative Humidity: Daily averages from weather stations.<br>Future Climate Projections: Daily surface temperature 2015–2100 from NEX-GDDP-CMIP6 (NASA), calibrated with historical data.<br>Heat Exposure: Temperatures above the minimum mortality temperature (MMT).	Temperature–mortality relationship: U-shaped, with increased risk above and below MMT (20.9–27.4 °C depending on subpopulation).<br>Extreme heat increased mortality risk for all causes and cardiovascular diseases; not significant for respiratory mortality.<br>Women had higher heat-related mortality than men; age differences inconclusive.<br>Projected heat-related mortality increases:<br>• 2050–2059: +1.3% (SSP2-4.5), +1.4% (SSP5-8.5).<br>• 2090–2099: +2.7% (SSP2-4.5), +4.7% (SSP5-8.5); equivalent to 3.1–4.9 deaths per 100 total deaths.<br>Vulnerable groups: women and adults >65. End-of-century increases under SSP2-4.5: women +3.5%, older adults +3.6%; under SSP5-8.5: women +6.1%, older adults +8.1%.<br>Heat-related cardiovascular mortality projected to rise significantly after mid-century, reaching +6% by end of century under SSP5-8.5.	8/8

(34)	Desing: case-crossover<br><br>Objetive: To evaluate the independent and joint association (interaction) between exposure to heat waves and PM2.5 levels on the risk of mortality from cardiovascular disease in Shenzhen, China, between 2013 and 2022.	N: 40.169<br>Age: ≤75 years/ >75<br>Sex (f/m): 15.216/24.953	Heat Exposure (Heat Waves): Defined by 90th, 92.5th, 95th percentiles of daily 24-hour average temperature for ≥2–8 consecutive days; data from CLDAS v2.0, 0.0625°×0.0625°; classified as heat wave days (1) or non-heat wave days (0).<br>$PM_{2.5}$ Exposure: Daily average from ChinaHighAirPollutant (CHAP) dataset, 1×1 km resolution; lag 0–1 day used.<br>Cardiovascular Disease (CVD) Mortality: ICD-10 I00–I99, including MI, ischemic heart disease, hemorrhage, heart failure; data from Shenzhen death surveillance system.	Analysis period: 2013–2022; 40,169 CVD death days vs. 136,415 control days; mean age 74, 62% men, ~50% >75 years.<br>On CVD death days: mean $PM_{2.5}$ = 27 μg/m³; mean temperature 13.8–30.5 °C.<br>Heat waves increased CVD mortality risk: e.g., 8-day 95th percentile heat wave → OR 1.91 (+91%).<br>$PM_{2.5}$: every 10 μg/m³ increase → 2.8–2.9% higher CVD death risk.<br>Combined heat waves + high $PM_{2.5}$ → higher CVD risk than either alone.<br>~2% of CVD deaths attributed to heat waves and/or high $PM_{2.5}$.<br>Older adults (>75) and women slightly more vulnerable, though not always statistically significant.	11/11
(37)	Desing: ecological observational and epidemiological modelling.<br><br>Objective: To assess the acute relationship between daily ambient temperature and emergency hospital admissions for dementia in older adults in	Age: 16-74 / 75-84/ ≥85 years	Daily Mean Ambient Temperature (°C): Met Office land stations and HadCET series.<br>Future Temperature Projections (RCP2.6, RCP8.5): UKCP18.<br>Emergency Hospital Admissions for Dementia (ICD F00–F03): NHS Digital.<br>Derived Variables: Admissions attributable to high temperatures, cumulative effects, lag-specific effects; analyzed with negative	Dementia admissions showed seasonality (summer and winter peaks), highest on Fridays.<br>Heat increased risk of admission above 17 °C (lowest risk at 14.6 °C); each 1 °C increase → +4.5% (95% CI: 2.9–6.1%).<br>Risk greater in older adults and disadvantaged groups:<br>• Age 16–74: +2.3%<br>• Age 75–84: +4.8%<br>• Age 85+: +4.8%<br>• Highest deprivation quintile: +4.8%<br>Regional effects observed nationwide; highest in Midlands and London.	11/11

	England, and to project the future burden of these admissions under climate change scenarios.		binomial regression, spline and cross-basis functions, fixed- and random-effects models, meta-analysis, log-linear extrapolation, Monte Carlo simulations.	Lag effects: same-day (lag 0) and 3–4 days later (lags 3–4); 1 °C above 17 °C → +1.8% same-day admissions.<br>Climate projections: average annual temperature increase 1.6–1.8 °C by 2040 (vs. 1981–2000).<br>Heat-attributable admissions projected to rise significantly:<br>• RCP2.6: +214% in 2030 (~357), +263% in 2040 (~412)<br>• RCP8.5: +194% in 2030 (~360), +294% in 2040 (~482)<br>• Largest increase in age 85+, up to +674% by 2040 under RCP8.5.	
(42)	Desing: ecological observational and epidemiological modelling.<br><br>Objetive: To evaluate the association between environmental temperature, especially extreme heat, and mortality in Spain during 1993–2013, and to analyse the effects of the implementation of the National Plan for the Prevention of Excess Temperature in Health (PNHP) on heat-attributable mortality.	N = 7.378.435<br>Age: 16-64 / 65-74 / 75-84 / > 85 years<br>Sex (f/m)	Total and Cause-Specific Mortality: Individual death records from Spanish National Statistics Institute (INE), ICD-coded.<br>Age and Sex: From INE death records.<br>Heat / Temperature Exposure: Daily maximum temperature from provincial capitals (European Climate Assessment & Dataset, ECA&D).<br>Heatwaves: Defined using provincial thresholds (PNHP) and historical temperature percentiles (≥2–4 consecutive days above 90th–97.5th percentiles).<br>Economic Vulnerability: Index from 2001 census: unemployment,	• Max temperature–mortality relationship: U-shaped; risk increased with both cold and hot temperatures.<br>• Cold-related mortality decreased from period 1 to 2 (33.6% → 14.7%; $p < 0.001$).<br>• Heat-related mortality risk slightly decreased at extreme temperatures (28.0% → 24.9%), with overlapping CIs.<br>• Overall temperature-attributable mortality declined (5.25% → 4.59%), mainly due to reduced cold-related deaths (4.26% → 2.88%).<br>• Extreme heat mortality slightly decreased (0.67% → 0.56%), moderate heat mortality increased (0.38% → 1.21%).<br>• Reductions in extreme cold most notable in >85 yrs, respiratory deaths, and rural areas; reductions in extreme heat more pronounced in older adults, cardiovascular deaths, and socioeconomically vulnerable populations.	11/11

			youth unemployment, temporary/unskilled workers, illiteracy.<br>Urban/Rural Environment: Municipality population ≥10,000 = urban; <10,000 = rural.<br>Air Conditioning: Percentage of households with AC from 2001 census.	• Geographic patterns: cold-related mortality higher in southern Spain/Mediterranean; decreases observed in most provinces except Madrid, Asturias, Murcia. Extreme heat mortality lower than cold-related, mainly affecting southern and central Spain; most provinces showed reductions, with slight increases in Ávila, Lérida, Navarra.	
(43)	Desing: time-series study<br><br>Objetive: To evaluate the relationship between temperature and all-cause mortality in people over 65 years of age in Spain, taking into account population ageing.	N= 10.791.306<br>Age: ≥ 65 years	All-Cause Mortality: Daily death microdata from National Statistics Institute (INE).<br>Chronological Age: Calculated from birth and death dates.<br>Prospective Age: Based on remaining life expectancy from INE life tables (smoothed with 11-year moving average).<br>Daily Mean Temperature: E-OBS v23.1 dataset, transformed into regionally weighted time series by population.<br>Relative Risk (RR) by Temperature: Quasi-Poisson models with DLNM and multivariate meta-analysis.	Mean daily temperature in Spain increased from 14.3 °C (1980–1994) to 15.5 °C (2004–2018); 99th percentile rose 26.5 °C → 27.1 °C.<br>Life expectancy increased: men +8.1 yrs (72.4→80.5), women +7.4 yrs (78.4→85.8). Prospective age gains greater in women (e.g., 65 yrs: men +3.5–5.2 yrs; 85 yrs: women +1.8–2.9 yrs).<br>RR of mortality from extreme temperatures higher in women, declined over time, especially middle-aged women; small differences between chronological and prospective age groups.<br>All-cause mortality higher in men, increased with age, decreased over time in chronological groups, stable in prospective groups.<br>Absolute risk of extreme heat deaths decreased: –54% women, –48% men; increased in ≥90 yrs in prospective groups (+1% men, +18% women).<br>Moderate heat: absolute risk decreased in chronological groups (–23% women, –16% men), increased in prospective groups (+46% women, +20% men).	9/11

				Average annual extreme heat deaths increased in older chronological groups; shift less pronounced in prospective groups.<br>Women had higher absolute risk and more heat-related deaths than men: recent period – 1,204/year (women) vs. 569/year (men).<br>Moderate heat deaths roughly doubled: women 1,448→2,896; men 580→998; female-to-male ratio increased from 2.5 to 2.9	

Note: Abbreviations: *AUC, area under the curve; BP, blood pressure; CVC, cutaneous vascular conductance; Δ, change in; ESQ-IV, Environmental Symptoms Questionnaire (version IV); f/m, female/male; HR, heart rate; HPLC, high-performance liquid chromatography; LBNP, lower body negative pressure; LC3-II, microtubule-associated protein 1A/1B-light chain 3 II; MAP, mean arterial pressure; MSNA, muscle sympathetic nerve activity; N_2, nitrogen gas; PECO, post-exercise circulatory occlusion; PBMCs, peripheral blood mononuclear cells; POMS-40, Profile of Mood States, 40-item version; PSI, Physiological Stress Index; RH, relative humidity; SBP, systolic blood pressure; SpO_2, peripheral oxygen saturation; Tdb, dry-bulb temperature; Tc / Tre / Tes, core body temperature (rectal / esophageal); Tmu, muscle temperature; Tsk, skin temperature; TS, thermal sensation; VO_2 / VO_2max, oxygen consumption / maximal oxygen uptake; WBGT, wet-bulb globe temperature; WBH, whole-body heating.*

Table S3. Observational and Epidemiological Studies: Characteristics and Main Findings

Study	Typology/Main objetive	Participants	Variables/Instruments	Main findings	International Banking Institute (JBI)
**(52)**	Desing: Analytical cross-sectional study<br><br>Objetive: To evaluate the impact of transient temperature changes during the process of getting up in winter on the thermal physiology	N= 35<br>N young adults: 5<br>N older adults: 30<br>Age young adults: 20-30 years	Outdoor & indoor air temperature (Ta) and relative humidity (RH): Thermometers & hygrometers<br>Cover temperature (Tcover): Thermometer under quilt<br>Black globe temperature (Tg): Black globe thermometer	Older adults showed a sharp increase in heart rate upon waking (from 68 to 88 bpm), followed by a drop to 76 bpm, with greater variability and amplitude than young adults, despite lower resting HR. 25% were at risk of tachycardia associated with temperature differences >15 °C, and HR variability increased with BMI, being higher in obese individuals.<br>Blood pressure showed small fluctuations, with 5% at risk of borderline hypertension, without significant	6/8

	and thermal perception of older adults, compared to young adults.	Age older adults: ≥60 years<br>Sex (f/m): 18/17	Heart rate (HR) & blood pressure (BP): Physiological monitoring wristband<br>Skin temperature (Tsk): Button thermometers on forehead, chest, arm, hand, thigh, foot<br>Core temperature (Tcore): In-ear thermometer<br>Thermal sensation & comfort: Questionnaires (-3 to +3 scale)<br>Thermal preference: Questionnaire (-1 to +1 scale)	influence from temperature differences. Core body temperature remained stable during getting up, although clothing thermal resistance was higher in older adults. Skin temperature in exposed extremities decreased more in older adults, particularly in hands and feet, indicating reduced thermal adaptation. Overall thermal perception was warm, with lower skin temperature sensitivity than young adults, who were more sensitive in chest and legs. Gender differences in local thermal perception were observed in young adults: men more sensitive in legs, women in chest. Thermal perception in older adults was weakly influenced by mean skin temperature, unlike the stronger relationship seen in young people.	
**(41)**	Desing: Analytical cross-sectional study<br><br>Objetive: Investigate skin temperature (Tsk) at rest and after acute exercise in older adults, and evaluate the influence of gender and cardiorespiratory fitness (CRF) on Tsk.	N: 92<br>Age: 65-75<br>Sex (f/m): 41/51	Skin Temperature (Tsk): Infrared thermography (FLIR E60), 25 ROIs.<br>Cardiorespiratory Fitness (CRF): Graded exercise test (modified Bruce protocol, Lode Valiant treadmill); VO2peak (ml/min, ml/kg/min).<br>Body Composition: Multi-frequency bioelectrical impedance (Tanita MC-780MA); body mass, BMI, fat mass, fat-free mass.<br>Anthropometry: Height (SECA 225).	Women had higher absolute/relative fat and lower CRF; BMI similar between sexes.<br>Men had higher resting Tsk in 19/25 ROIs; largest differences in posterior thigh (+0.91 °C), posterior calf (+0.90 °C), posterior arm (+0.80 °C), anterior thigh (+0.60 °C). Most differences remained after adjusting for fat mass and VO2peak, except chin.<br>After exercise: distal upper extremities (hands/fingers) increased in temperature; proximal upper extremities (arms/torso) decreased; lower extremities increased; core stable. Men had larger forearm temperature decreases than women; differences in posterior arm, posterior leg, anterior thigh remained significant after adjustment.<br>Higher VO2peak associated with greater lower extremity temperature increases in both sexes; men	8/8

				had lower proximal upper extremity temperature, women had lower core and higher distal upper extremity temperatures. Similar patterns observed using time to exhaustion.	
**(49)**	Desing: case-crossover<br><br>Objetive: Evaluate the intra-seasonal variation (beginning vs. end of the warm season) in the association between exposure to high ambient temperatures and the risk of mortality, and identify population subgroups that are most vulnerable to extreme heat.	N: 1.132.980<br>Age: ≤64 / 65–74 / ≥75 years<br>Sex (f/m): 479.121/ 653.859	Daily Temperature, Relative Humidity, Wind Speed: China Meteorological Data Network (0.01° × 0.01° resolution).<br>Daily $PM_{2.5}$ Concentration: Validated predictive model (0.01° × 0.01°).<br>Mortality Data (all-cause and cause-specific, ICD-10): Official Shandong CDC registry, including date of death, age, sex, education, residential address.<br>Mortality Risk Estimation: Conditional logistic regression with distributed lag non-linear models (DLNM), OR at 97.5th percentile vs. minimum mortality temperature (MMT).	Warm season (June–September) daily temperature averaged 24.1 ± 3.3 °C; extreme heat defined as >36.6 °C.<br>1,132,980 deaths recorded in 1,822 subdistricts (2013–2018).<br>J-shaped relationship between temperature and mortality; MMT = 22.5 °C.<br>Extreme heat increased mortality risk: cumulative OR 3.41 (95% CI: 3.11–3.74) over 0–10 days; effect strongest on same day (lag 0), decreasing to day 6, with mortality deficit until day 9.<br>Risk decreased across the season: early warm season OR 3.52 → late warm season OR 2.85 (p = 0.028).<br>Vulnerable groups: women (OR 4.03 vs. 3.00 for men, p = 0.002), adults >75 years (OR 5.06 vs. 2.14 for <65, p < 0.001), lower education (OR 3.43 vs. 1.79 for higher education, p = 0.003).<br>Major causes of heat-related mortality: cardiovascular (OR 5.35) and respiratory diseases (OR 4.73).<br>No significant urban–rural differences (OR 3.23 vs. 3.47, p = 0.450).	11/11
**(48)**	Desing: ecological observational time series<br><br>Objetive: Estimate heat-attributable mortality in	N= 53.964<br>Age: < 65 years / > 65 years	Daily Cause-Specific Mortality: Daily counts by cause from Cyprus Ministry of Health (Health Monitoring Unit),	Temperature–mortality relationship: U-shaped, with increased risk above and below MMT (20.9–27.4 °C depending on subpopulation).	8/8

	Cyprus for future decades of the 21st century under moderate (SSP2-4.5) and extreme (SSP5-8.5) climate scenarios, based on the observed relationship between ambient temperature and mortality in the period 2004–2019.	Sex (f/m): 26.331/27.633	including date, sex, age, cause of death (ICD-10).<br>Daily Ambient Temperature: Mean of daily minimum and maximum from 7 Cyprus Meteorology Department stations.<br>Daily Relative Humidity: Daily averages from weather stations.<br>Future Climate Projections: Daily surface temperature 2015–2100 from NEX-GDDP-CMIP6 (NASA), calibrated with historical data.<br>Heat Exposure: Temperatures above the minimum mortality temperature (MMT).	Extreme heat increased mortality risk for all causes and cardiovascular diseases; not significant for respiratory mortality.<br>Women had higher heat-related mortality than men; age differences inconclusive.<br>Projected heat-related mortality increases:<br>2050–2059: +1.3% (SSP2-4.5), +1.4% (SSP5-8.5).<br>2090–2099: +2.7% (SSP2-4.5), +4.7% (SSP5-8.5); equivalent to 3.1–4.9 deaths per 100 total deaths.<br>Vulnerable groups: women and adults >65. End-of-century increases under SSP2-4.5: women +3.5%, older adults +3.6%; under SSP5-8.5: women +6.1%, older adults +8.1%.<br>Heat-related cardiovascular mortality projected to rise significantly after mid-century, reaching +6% by end of century under SSP5-8.5.	
**(47)**	Desing: case-crossover<br><br>Objetive: To evaluate the independent and joint association (interaction) between exposure to heat waves and PM2.5 levels on the risk of mortality from cardiovascular disease in Shenzhen, China, between 2013 and 2022.	N: 40.169<br>Age: ≤75 years/ >75<br>Sex (f/m): 15.216/24.953	Heat Exposure (Heat Waves): Defined by 90th, 92.5th, 95th percentiles of daily 24-hour average temperature for ≥2–8 consecutive days; data from CLDAS v2.0, 0.0625°×0.0625°; classified as heat wave days (1) or non-heat wave days (0).<br>$PM_{2.5}$ Exposure: Daily average from ChinaHighAirPollutant (CHAP) dataset, 1×1 km resolution; lag 0–1 day used.<br>Cardiovascular Disease (CVD) Mortality: ICD-10 I00–I99, including MI, ischemic heart	Analysis period: 2013–2022; 40,169 CVD death days vs. 136,415 control days; mean age 74, 62% men, ~50% >75 years.<br>On CVD death days: mean $PM_{2.5}$ = 27 μg/m³; mean temperature 13.8–30.5 °C.<br>Heat waves increased CVD mortality risk: e.g., 8-day 95th percentile heat wave → OR 1.91 (+91%).<br>$PM_{2.5}$: every 10 μg/m³ increase → 2.8–2.9% higher CVD death risk.<br>Combined heat waves + high $PM_{2.5}$ → higher CVD risk than either alone.<br>~2% of CVD deaths attributed to heat waves and/or high $PM_{2.5}$.<br>Older adults (>75) and women slightly more vulnerable, though not always statistically significant.	11/11

			disease, hemorrhage, heart failure; data from Shenzhen death surveillance system.		
**(46)**	Desing: ecological observational and epidemiological modelling.<br><br>Objetive: To assess the acute relationship between daily ambient temperature and emergency hospital admissions for dementia in older adults in England, and to project the future burden of these admissions under climate change scenarios.	Age: 16-74 / 75-84/ ≥85 years	Daily Mean Ambient Temperature (°C): Met Office land stations and HadCET series.<br>Future Temperature Projections (RCP2.6, RCP8.5): UKCP18.<br>Emergency Hospital Admissions for Dementia (ICD F00–F03): NHS Digital.<br>Derived Variables: Admissions attributable to high temperatures, cumulative effects, lag-specific effects; analyzed with negative binomial regression, spline and cross-basis functions, fixed- and random-effects models, meta-analysis, log-linear extrapolation, Monte Carlo simulations.	Dementia admissions showed seasonality (summer and winter peaks), highest on Fridays.<br>Heat increased risk of admission above 17 °C (lowest risk at 14.6 °C); each 1 °C increase → +4.5% (95% CI: 2.9–6.1%).<br>Risk greater in older adults and disadvantaged groups:<br>Age 16–74: +2.3%<br>Age 75–84: +4.8%<br>Age 85+: +4.8%<br>Highest deprivation quintile: +4.8%<br>Regional effects observed nationwide; highest in Midlands and London.<br>Lag effects: same-day (lag 0) and 3–4 days later (lags 3–4); 1 °C above 17 °C → +1.8% same-day admissions.<br>Climate projections: average annual temperature increase 1.6–1.8 °C by 2040 (vs. 1981–2000).<br>Heat-attributable admissions projected to rise significantly:<br>RCP2.6: +214% in 2030 (~357), +263% in 2040 (~412)<br>RCP8.5: +194% in 2030 (~360), +294% in 2040 (~482)<br>Largest increase in age 85+, up to +674% by 2040 under RCP8.5.	11/11
**(45)**	Desing: ecological observational and epidemiological modelling.	N = 7.378.435<br>Age: 16-64 / 65-74 / 75-84 / > 85 years<br>Sex (f/m)	Total and Cause-Specific Mortality: Individual death records from Spanish National Statistics Institute (INE), ICD-coded.	Max temperature–mortality relationship: U-shaped; risk increased with both cold and hot temperatures.<br>Cold-related mortality decreased from period 1 to 2 (33.6% → 14.7%; p < 0.001).	11/11

	Objetive: To evaluate the association between environmental temperature, especially extreme heat, and mortality in Spain during 1993–2013, and to analyse the effects of the implementation of the National Plan for the Prevention of Excess Temperature in Health (PNHP) on heat-attributable mortality.		Age and Sex: From INE death records.<br>Heat / Temperature Exposure: Daily maximum temperature from provincial capitals (European Climate Assessment & Dataset, ECA&D).<br>Heatwaves: Defined using provincial thresholds (PNHP) and historical temperature percentiles (≥2–4 consecutive days above 90th–97.5th percentiles).<br>Economic Vulnerability: Index from 2001 census: unemployment, youth unemployment, temporary/unskilled workers, illiteracy.<br>Urban/Rural Environment: Municipality population ≥10,000 = urban; <10,000 = rural.<br>Air Conditioning: Percentage of households with AC from 2001 census.	Heat-related mortality risk slightly decreased at extreme temperatures (28.0% → 24.9%), with overlapping CIs.<br>Overall temperature-attributable mortality declined (5.25% → 4.59%), mainly due to reduced cold-related deaths (4.26% → 2.88%).<br>Extreme heat mortality slightly decreased (0.67% → 0.56%), moderate heat mortality increased (0.38% → 1.21%).<br>Reductions in extreme cold most notable in >85 yrs, respiratory deaths, and rural areas; reductions in extreme heat more pronounced in older adults, cardiovascular deaths, and socioeconomically vulnerable populations.<br>Geographic patterns: cold-related mortality higher in southern Spain/Mediterranean; decreases observed in most provinces except Madrid, Asturias, Murcia.<br>Extreme heat mortality lower than cold-related, mainly affecting southern and central Spain; most provinces showed reductions, with slight increases in Ávila, Lérida, Navarra.	
**(44)**	Desing: time-series study<br><br>Objetive: To evaluate the relationship between temperature and all-cause mortality in people over 65 years of age in Spain,	N= 10.791.306<br>Age: ≥ 65 years	All-Cause Mortality: Daily death microdata from National Statistics Institute (INE).<br>Chronological Age: Calculated from birth and death dates.<br>Prospective Age: Based on remaining life expectancy from	Mean daily temperature in Spain increased from 14.3 °C (1980–1994) to 15.5 °C (2004–2018); 99th percentile rose 26.5 °C → 27.1 °C.<br>Life expectancy increased: men +8.1 yrs (72.4→80.5), women +7.4 yrs (78.4→85.8). Prospective age gains greater in women (e.g., 65 yrs: men +3.5–5.2 yrs; 85 yrs: women +1.8–2.9 yrs).	9/11

taking into account population ageing.	INE life tables (smoothed with 11-year moving average).<br>Daily Mean Temperature: E-OBS v23.1 dataset, transformed into regionally weighted time series by population.<br>Relative Risk (RR) by Temperature: Quasi-Poisson models with DLNM and multivariate meta-analysis.	RR of mortality from extreme temperatures higher in women, declined over time, especially middle-aged women; small differences between chronological and prospective age groups.<br>All-cause mortality higher in men, increased with age, decreased over time in chronological groups, stable in prospective groups.<br>Absolute risk of extreme heat deaths decreased: –54% women, –48% men; increased in ≥90 yrs in prospective groups (+1% men, +18% women).<br>Moderate heat: absolute risk decreased in chronological groups (–23% women, –16% men), increased in prospective groups (+46% women, +20% men).<br>Average annual extreme heat deaths increased in older chronological groups; shift less pronounced in prospective groups.<br>Women had higher absolute risk and more heat-related deaths than men: recent period – 1,204/year (women) vs. 569/year (men).<br>Moderate heat deaths roughly doubled: women 1,448→2,896; men 580→998; female-to-male ratio increased from 2.5 to 2.9

Note: Abbreviations: *AC, air conditioning; BMI, body mass index; BP, blood pressure; CDC, Centers for Disease Control; CVD, cardiovascular disease; CRF, cardiorespiratory fitness; DLNM, distributed lag non-linear model; ECA&D, European Climate Assessment & Dataset; f/m, female/male; FLIR, forward-looking infrared; HR, heart rate; ICD, International Classification of Diseases; MMT, minimum mortality temperature; MI, myocardial infarction; NEX-GDDP-CMIP6, NASA Earth Exchange Global Daily Downscaled Projections – Coupled Model Intercomparison Project Phase 6; NHS, National Health Service; OR, odds ratio; PNHP, National Plan for the Prevention of Excess Temperature in Health;* $PM_{2.5}$*, particulate matter ≤2.5 µm; RCP, Representative Concentration Pathway; RR, relative risk; ROIs, regions of interest; SSP, Shared Socioeconomic Pathway; Ta, air temperature; Tcover, cover temperature; Tg, black globe temperature; Tcore, core body temperature; Tsk, skin temperature; UKCP18, UK Climate Projections 2018;* VO_2*peak, peak oxygen consumption; Δ, change in; µg/*m^3*, micrograms per cubic meter.*
